# Solution structure of the isolated histone H2A-H2B heterodimer

**DOI:** 10.1038/srep24999

**Published:** 2016-05-16

**Authors:** Yoshihito Moriwaki, Tsutomu Yamane, Hideaki Ohtomo, Mitsunori Ikeguchi, Jun-ichi Kurita, Masahiko Sato, Aritaka Nagadoi, Hideaki Shimojo, Yoshifumi Nishimura

**Affiliations:** 1 Graduate School of Medical Life Science, Yokohama City University, 1-7-29 Suehiro-cho, Tsurumi-ku, Yokohama 230-0045, Japan

## Abstract

During chromatin-regulated processes, the histone H2A-H2B heterodimer functions dynamically in and out of the nucleosome. Although detailed crystal structures of nucleosomes have been established, that of the isolated full-length H2A-H2B heterodimer has remained elusive. Here, we have determined the solution structure of human H2A-H2B by NMR coupled with CS-Rosetta. H2A and H2B each contain a histone fold, comprising four α-helices and two β-strands (α_1_–β_1_–α_2_–β_2_–α_3_–α_C_), together with the long disordered N- and C-terminal H2A tails and the long N-terminal H2B tail. The N-terminal α_N_ helix, C-terminal β_3_ strand, and 3_10_ helix of H2A observed in the H2A-H2B nucleosome structure are disordered in isolated H2A-H2B. In addition, the H2A α_1_ and H2B α_C_ helices are not well fixed in the heterodimer, and the H2A and H2B tails are not completely random coils. Comparison of hydrogen-deuterium exchange, fast hydrogen exchange, and {^1^H}-^15^N hetero-nuclear NOE data with the CS-Rosetta structure indicates that there is some conformation in the H2A 3_10_ helical and H2B Lys11 regions, while the repression domain of H2B (residues 27–34) exhibits an extended string-like structure. This first structure of the isolated H2A-H2B heterodimer provides insight into its dynamic functions in chromatin.

In eukaryotes, DNA is stably stored in the highly ordered structure chromatin. The fundamental repeating structural unit within chromatin is the nucleosome, which comprises approximately 146 bp of DNA wrapped around a histone octamer, containing two dimers of H2A-H2B and one tetramer of (H3-H4)_2_^1^. The nucleosome is assembled in a stepwise manner: a tetramer of (H3-H4)_2_ is first deposited on the central part of DNA; two heterodimers of H2A-H2B are then added to the peripheral parts of DNA, and the nucleosome is completed^2^. During gene transcription, H2A-H2B seems to dynamically detach from and assemble on the nucleosome with the aid of histone chaperones^3^, and the isolated H2A-H2B heterodimer is structurally stable. A structural comparison of the isolated H2A-H2B and its counterpart in the nucleosome would help to elucidate both the function of various histone chaperones and nucleosome dynamics^4^.

To date, the structure of H2A-H2B dimer alone has not been solved, whereas two structures of the H2A-H2B dimer within the nucleosome have been available for some time^5^,^6^,^7^,^8^,^9^. In addition, structures of H2A-H2B or H2A.Z-H2B with a histone chaperone are available; however, the tail of histone H2A or H2A.Z and that of H2B are not present in these complexes, for example, FACT Spt16M linked to H2B(amino acids 24–122)–H2A(13–106)^10^, Chz1 bound to H2B(37–131) linked to H2A.Z(29–125)^11^, Swr1 bound to H2B(36–130) linked to H2A.Z(22–118)^12^ and ANP32E bound to H2B(30–125)–H2A.Z(18–127)^13^. Histone tails play important roles in the dynamic functions of chromatin through posttranslational modifications such as acetylation, phosphorylation and methylation^14^,^15^,^16^,^17^,^18^,^19^.

Here, we have determined the tertiary structure of the full-length H2A-H2B in isolation by NMR coupled with the CS-Rosetta procedure^20^,^21^. The isolated H2A-H2B heterodimer comprises 256 amino acids with long disordered tails, which is big enough for the CS-Rosetta calculation^22^; as a result, the Rosetta protocols, AbinitioRelax^23^ and FloppyTail^24^, were used to obtain structures of H2A-H2B in isolation. The calculated structures show that both histones contain a four-helix core, arranged as α_1_–β_1_–α_2_–β_2_–α_3_–α_C_, similar to their corresponding structures in the nucleosome. Outside the core region of H2A, however, the N-terminal α_N_ helix, C-terminal β_3_ strand and 3_10_ helix that are present in the nucleosome are entirely disordered in the isolated H2A-H2B, instead becoming long disordered tails of about 30 amino acids at both the N- and C-termini. The α_N_ helix, β_3_ strand and 3_10_ helix of H2A are stabilized in the nucleosome by interactions with DNA or histones H3-H4. Without these interactions, the α_N_ helix, β_3_ strand and 3_10_ helix regions become disordered in the isolated H2A-H2B dimer.

Furthermore, the calculated structures of H2A-H2B indicate that the positions of the H2A α_1_ and H2B α_C_ helices are not well fixed as compared with other helices, suggesting that these two helices dynamically fluctuate in solution. To reveal the dynamics of H2A-H2B, we performed hydrogen-deuterium (H/D) exchange^25^, fast hydrogen exchange^26^ and {^1^H}-^15^N hetero-nuclear NOE^27^ experiments on H2A-H2B. Comparison of these data with the calculated structures suggests that the long disordered tails of H2A-H2B form some dynamic conformations.

## Results

### Secondary structural elements of the isolated H2A-H2B heterodimer

**Figure 1:**
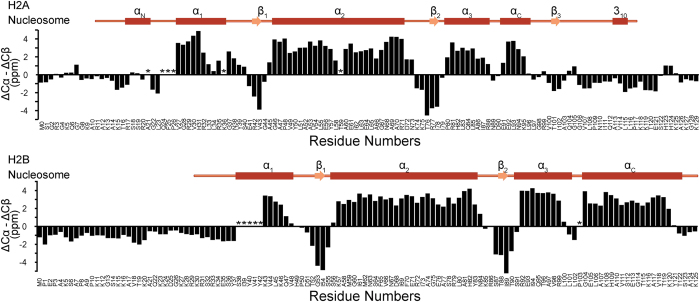
Secondary structures of the isolated H2A-H2B heterodimer. The secondary structures, chemical shift indices and amino acid sequences of H2A-H2B are shown. The bar graph indicates the chemical shift indices of H2A (top) and H2B (bottom) in the H2A-H2B heterodimer based on the Cα and Cβ chemical shifts. Asterisks indicate unassigned residues in the NMR spectra. The corresponding secondary structural elements in the nucleosome crystal structure are indicated as boxes (α-helices) and arrows (β-strands).

We examined the secondary structures of the isolated H2A-H2B heterodimer by TROSY-NMR (Fig. 1). Almost all of the main-chain signals (i.e., HN, N, Cα, Cβ and C’ signals) could be assigned (96% for H2A; 95% for H2B). The main-chain signals of Ala21, Gln24, Phe25 Pro26, Lys36 and Thr59 of H2A and Ser38, Ile39, Tyr40 Val41, Tyr42 and Pro103 of H2B could not be assigned. As shown in the experimental chemical shift indices obtained from the Cα and Cβ chemical shift values, both H2A and H2B contain a core histone fold comprising four α-helices together with two β-strands—namely, α_1_–β_1_–α_2_–β_2_–α_3_–α_C_, much as is observed for their counterparts in the nucleosome. Outside the histone fold of H2A, however, the N-terminal α_N_ helix and the C-terminal β_3_ strand and 3_10_ helix observed in the nucleosome are entirely disordered in the isolated H2A-H2B heterodimer (Fig. 1). These structural elements of H2A are stabilized in the nucleosome: the α_N_ helix by hydrogen bonding to DNA; the β_3_ strand by forming a β-sheet with H4 β_3_; and the 3_10_ helix by interaction with the α_2_ helix of H3 and the L1 loop of H4.

### Modeled solution structures of the isolated H2A-H2B heterodimer

**Figure 2:**
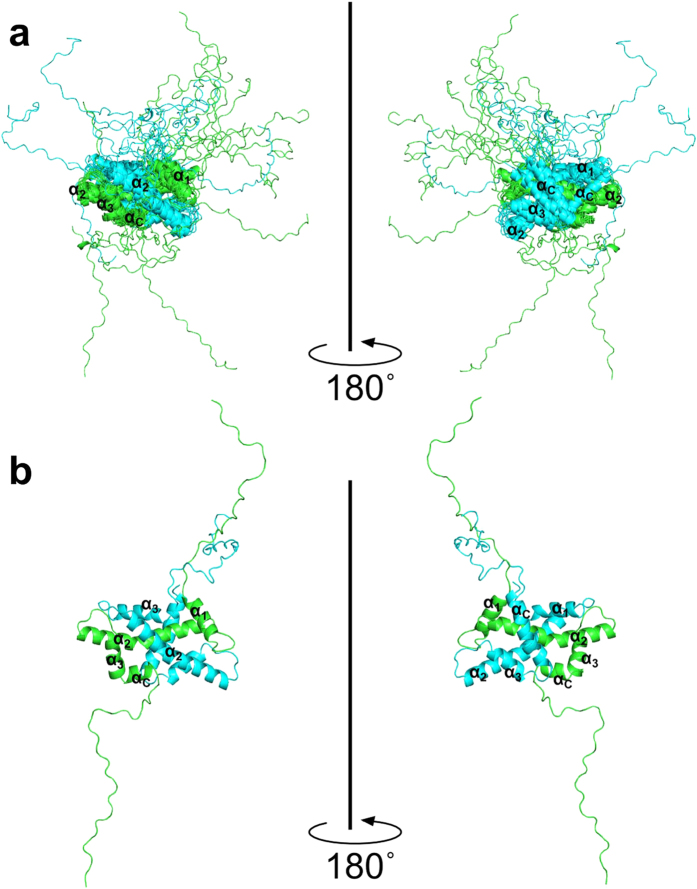
Structural analysis of the H2A-H2B heterodimer. (**a**) Ten model structures of the isolated H2A-H2B heterodimer. (**b**) The structure with the lowest CS-Rosetta energy score of core region. H2A and H2B are shown in green and cyan, respectively. Each secondary structural element is labeled. The image was drawn with PyMol^39^.

**Figure 3:**
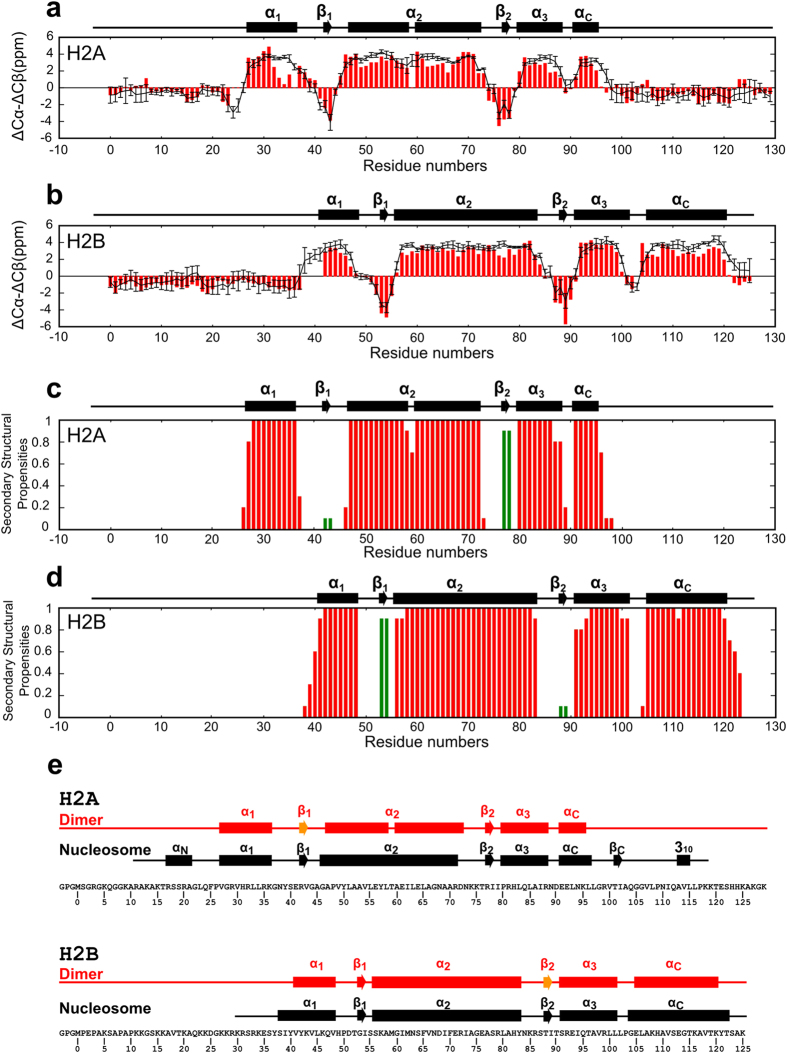
Observed and calculated chemical shift indices and secondary structural propensities of the isolated H2A-H2B heterodimer, and comparison with the nucleosome structure. (**a**,**b**) Comparison between the calculated (black line with error bars) and observed (red bars) values of chemical shift indices for H2A (**a**) and H2B (**b**). The secondary structural elements are indicated as boxes (α-helices) and arrows (β-strands). (**c**,**d**) Secondary structural propensities of the modeled structures of H2A (**c**) and H2B (**d**) in the isolated H2A-H2B heterodimer. Secondary structures were calculated with the DSSP program^29^: α-helix (“H” in DSSP) and β-strand (“B” and “E” in DSSP) residues are indicated by red and green bars, respectively. (**e**) Secondary structures of H2A-H2B in the isolated heterodimer (red) and the nucleosome core (PDB_ID: 3AFA chain c and d, colored by black). The secondary structural regions in the isolated heterodimer H2A-H2B were defined on the basis of secondary structural propensities in (**c**,**d**) of more than 0.8. Only the H2A β_1_ and H2B β_2_ secondary structural propensities were less than 0.8 (orange).

The 10 modeled structures of H2A-H2B are shown in Fig. 2 and the Ramachandran plots of these structures are shown in Supplementary Fig. S1. The convergence of these models in terms of Rosetta energy, Cα-RMSD and 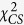 is shown in Supplementary Figs S2–S8. The chemical shift values calculated from the model structures generated by the CS-Rosetta-AbinitioRelax and FloppyTail protocols were well converged with the observed values. As shown in Supplementary Fig. S9, the averages of the chemical shift values of Cα and Cβ calculated from the model structures were in good agreement with the experimental chemical shift values. The calculated chemical shift indices of the model structures were also in good agreement with the experimental values (Fig. 3a,b), and consistent with the secondary structural propensities of the modeled structures (Fig. 3c,d). The tertiary structural arrangement of the model structures was close to that of the histone dimer in the nucleosome crystal structure (Fig. 3e).

**Figure 4:**
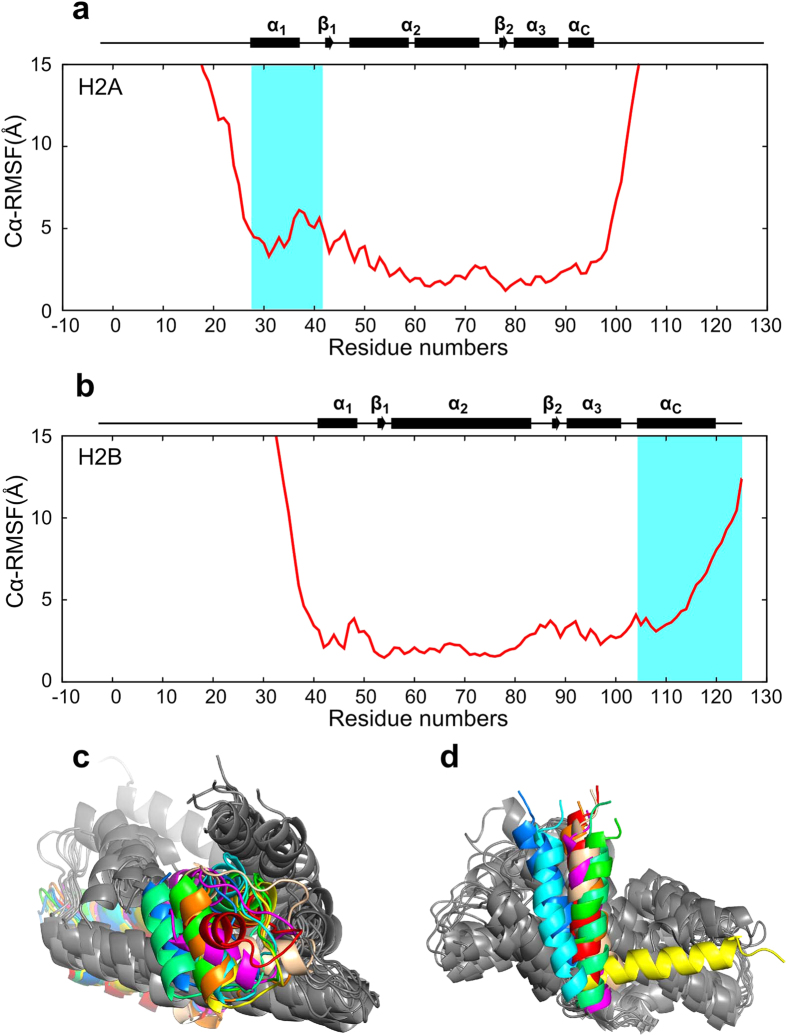
Fluctuations of the modeled structures of the isolated H2A-H2B heterodimer. (**a**,**b**) Cα root mean square fluctuations (RMSF) of H2A (**a**) and H2B (**b**) in the solution structures of the isolated H2A-H2B heterodimer. The structures were aligned via the core region of H2A-H2B. The flexible regions, from α_1_ to β_1_ of H2A (**a**), and α_C_ of H2B (**b**), are shown in cyan. The secondary structural elements are indicated as boxes (α-helices) and arrows (β-strands). (**c**,**d**) The 10 structures of the core region of the isolated H2A-H2B heterodimer. The flexible regions of H2A (**c**) and H2B (**d**) are shown in color, and correspond to the cyan region in (**a**,**b**), respectively. The images were drawn with PyMol^39^.

Although the core region of the solution structures was similar to the core structure in the nucleosome, α_1_ and the following loop connecting α_1_ and α_2_ (the L1 loop) in H2A were not well fixed in the solution structures (Fig. 4a,c). In addition, the location of the α_c_ helix in H2B was not fixed in the solution structures (Fig. 4b,d). In the nucleosome, the H2A α_1_ and L1 loop region are bound to DNA and the other H2A subunit^28^ (See Supplementary Fig. S10), and these interactions seem to fix the location of the α_1_ helix and L1 loop, as compared with the isolated H2A-H2B dimer. In the nucleosome, the α_c_ helix of H2B is stabilized by the α_N_ helix of H2A and denaturation of the H2A α_N_ helix in the isolated heterodimer leads to a random orientation of the H2B α_c_ helix (See Supplementary Fig. S10).

### Structural properties of the flexible tails of the modeled solution structures of the isolated H2A-H2B heterodimer

As judged by the DSSP program^29^, the three long flexible tails (H2A N- and C-termini, and H2B N-terminus) are random coil structures, which is consistent with the observed chemical shift values (Fig. 1). In addition, the backbone accessible surface areas show that the three long flexible tails are almost completely exposed to solvent (See Supplementary Fig. S11). As shown in Supplementary Figs S12 and S13, most of residues in the tails had no secondary structure; however, bend and turn structures defined by DSSP were observed in the models, suggesting that the H2A and H2B tails are not completely random coils. The average distances between Cα_*i−*4_ and Cα_*i*+4_, *d*(Cα_*i*−4_, Cα_*i*+4_) (where *i* is the residue number) were as low as ~15 Å, indicating bending structures in the tails (See Supplementary Fig. S12). For reference, the *d*(Cα_*i*−4_, Cα_*i*+4_) values of α-helical and β-strand elements are ~11 and ~28 Å, respectively. It should be noted that there were some bend and/or turn structures in two regions of the H2A N-terminal tail (Met0-Lys9 and Thr16-Phe25), three regions of the H2A C-terminal tail (Ile102-Val107, Pro109-Val114 and Ser122-Lys127) and two regions of the H2B N-terminal tail (Met0-Lys20 and Leu23-Lys30). Thus, even the disordered regions have some secondary structural propensity and can be distinguished by the dynamics of H2A-H2B as shown below.

### H/D exchange experiments

**Figure 5:**
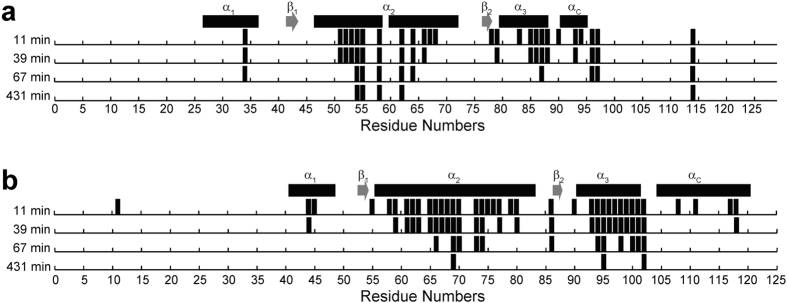
H/D exchange in the isolated H2A-H2B heterodimer. The lyophilized H2A-H2B sample was reconstituted in D_2_O and 2D TROSY-^1^H-^15^N HSQC spectra were recorded at 11, 39, 67 and 431 minutes. Identifiable signals of amino acids in H2A (**a**) and H2B (**b**) in each spectra are indicated by bars. The secondary structures determined by CS-Rosetta are indicated as boxes (α-helices) and arrows (β-strands).

To reveal the dynamic character of H2A-H2B, we performed H/D exchange, fast hydrogen exchange and {^1^H}-^15^N hetero-nuclear NOE experiments by NMR. Eleven minutes after reconstitution of the lyophilized H2A-H2B sample into D_2_O, amide signals were still observed for Leu34, Leu51, Ala52, Ala53, Val54, Leu55, Leu58, Ile62, Glu64, Ala66, Gly67, Asn68, Ile78, Ile79, Leu83, Leu85, Ala86, Ile87, Arg88, Asp90, Leu93, Asn94, Leu96, Leu97 and Val114 in H2A and for Lys11, Val44, Leu45, Ser55, Ala58, Met59, Ile61, Met62, Asn63, Phe65,Val66, Asn67, Asp68, Ile69, Phe70, Ile73, Ala74, Gly75, Glu76, Ala77, Arg79, Leu80, Arg86, Thr90, Glu93, Ile94, Gln95, Thr96, Ala97, Val98, Arg99, Leu100, Leu101, Leu102, Lys108, Val111, Ala117 and Val118 in H2B (Fig. 5).

Notably, almost all amide signals of residues in the N-terminal tail and the α_1_–β_1_ regions of H2A except that of Leu34 had disappeared by 11 minutes after solvation in D_2_O, suggesting that these regions are very flexible. In contrast, the amide signals of residues in the core α_2_, α_3_ and α_C_ helices of H2A were still present even after 39 minutes, suggesting that these regions form a rigid structure in H2A-H2B. Surprisingly, the amide signal of Val114 in the H2A C-terminal tail exhibited slower exchange as compared with that of the surrounding amino acids, suggesting that this hydrophobic residue in the disordered tail may form a somewhat unusual conformation, protecting the amide from solvent exchange.

The amide protons of the α_2_ and α_3_ helices of H2B were well protected from water exchange, suggesting these two helices form a rigid structure; however the amide protons of the α_1_ and α_C_ helices disappeared relatively rapidly, suggesting that these two helices fluctuate in solution. The amide proton of Lys11 in the H2B N-terminal tail exhibited relatively slow exchange as compared with other amide protons in the H2B N-terminal tail region. This lysine residue at position 11 may interact with other residues to protect the amide proton against solvent exchange via unusual conformations formed in the disordered N-terminal tails.

### HET^ex^-BEST-TROSY experiments

**Figure 6:**
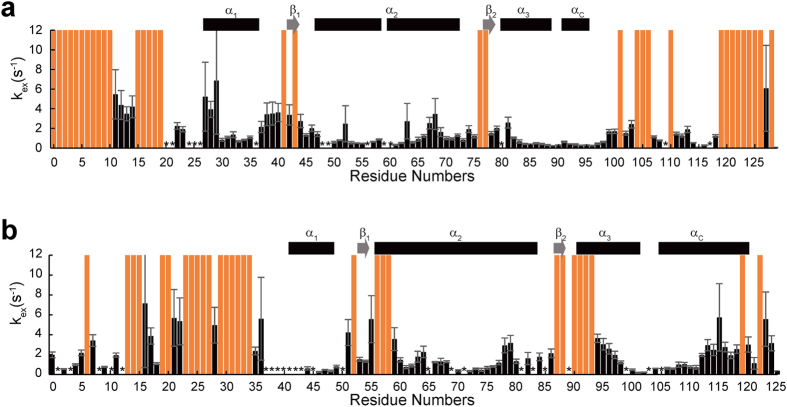
Fast exchange in the isolated H2A-H2B heterodimer. Bars show the exchange rates, obtained by HET^ex^-BEST-TROSY, of amide protons in the main chain of H2A (**a**) and H2B (**b**) with water molecules. Asterisks indicate signals of amino that were not assigned by NMR. Red bars indicate faster exchange rates (*k*_*ex*_ over 10 s^−1^). The secondary structures determined by CS-Rosetta are indicated as boxes (α-helices) and arrows (β-strands).

By using HET^ex^ -BEST-TROSY, we could monitor rapid exchange of the amide protons with water in the time range of 0.1 s^−1^ < *k*_*ex*_ < 10 s^−1^. The amide protons of the α_1_, α_2_, α_3_ and α_C_ helices of both H2A and H2B showed slow exchange with water as compared with the N-terminal and C-terminal tails of H2A and the N-terminal tail of H2B (Fig. 6). By contrast, the amide protons of an H2A C-terminal region comprising Val107, Leu108, Ile111, Gln112, Ala113, Val114, Leu115, Leu116 and Lys118 showed relatively slow exchange. Notably, the region of slow exchange with water contains Val114, the amide proton of which showed slow H/D exchange. Thus, this region seems to form unusual conformations that protect these amide protons against solvent.

In addition, an H2A N-terminal tail region comprising Arg11, Ala12, Lys13 and Ala14 showed relatively slower exchange with water, again suggesting the formation of some conformations that protect the amide protons. In addition, an H2B N-terminal region comprising Ala4, Lys5, Ala7, Ala9 and Lys11 showed relatively slow exchange behavior. This region contains Lys11, the amide proton of which showed slow H/D exchange. In HET^ex^-BEST-TROSY experiments, the OH group of a serine or threonine residue decreases the intensity of a nearby amide proton by exchange relayed NOE. This may account for the larger *k*_*ex*_ values observed for Glu41 and Leu63 of H2A and Thr52, Ser55, Ala58, Met59, Thr90, Arg92, Glu93 and Thr119 of H2B as compared with the *k*_*ex*_ value of the surrounding amino acids. The amide protons of Val27, Gly28, Arg29, Val43, Thr76 and Arg77 of H2A and Ser56, Lys57, Ser87, Thr88 and Ser91 of H2B are exposed to solvent; therefore, the *k*_*ex*_ values of these residues are large.

### Hetero-nuclear NOE experiments

**Figure 7:**
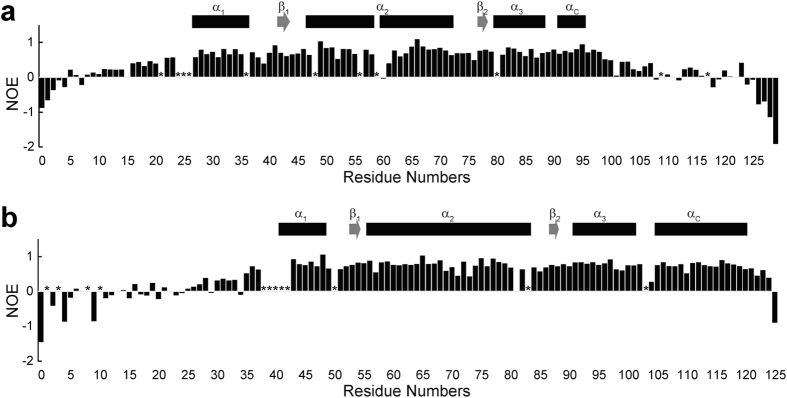
Hetero-nuclear NOE data for the isolated H2A-H2B heterodimer. The {^1^H}-^15^N hetero-nuclear NOE values of NH signals are shown by bars. Asterisks indicate signals of amino acids that were not assigned by NMR. The secondary structures determined by CS-Rosetta indicated as boxes (α-helices) and arrows (β-strands).

The hetero-nuclear NOE values, which represent backbone dynamics on the picosecond to nanosecond timescale, showed that the 7 N-terminal and 11 C-terminal residues of H2A and the 17 N-terminal and 1 C-terminal residues of H2B were negative, indicating that these terminal regions dynamically fluctuate on this timescale, adopting random coil structures. The hetero-nuclear NOE spectra also showed relatively high values over 0.5 for the core regions of H2A (Val27-Lys95) and H2B (Lys43-Lys120), with the NOE values falling to negative values for residues in both the N-termini and C-termini of H2A and H2B (Fig. 7). In the N-termini of both H2A and H2B, however, slightly positive values were observed for amino acids Gly8-Leu23 of H2A and amino acids of Asp25-Tyr37 of H2B, suggesting that these regions adopt a somewhat rigid character in the disordered tails. Furthermore, a C-terminal region of H2A (amino acids Leu96–Leu116) also seems to adopt a somewhat rigid character with slightly positive NOE values. Notably, these regions roughly correspond to the region of slow exchange with water.

## Discussion

Histone proteins have N-terminal and/or C-terminal flexible tails, which are modified by methylation and acetylation, and influence chromatin remodeling^14^,^15^,^16^,^17^,^18^,^19^. In the crystal structure of the nucleosome, these histone tails are not observed owing to their flexibility^5^,^6^,^7^,^8^,^9^. Here we have solved the whole structure of the isolated H2A-H2B heterodimer including its flexible tails. In solution, the isolated H2A-H2B dimer was revealed to have the histone fold structure: both histones contain a four-helix core, namely, α_1_–β_1_–α_2_–β_2_–α_3_–α_C_, similar to their counterpart structures in the nucleosome. Outside the core of H2A, by contrast, the N-terminal α_N_ helix and the C-terminal β_3_ strand and 3_10_ helix of H2A observed in the nucleosome are entirely disordered in the isolated H2A-H2B dimer, resulting in long disordered tails of about 30 amino acids at both N- and C-termini. In both histone folds, the locations of the H2A α_1_ and H2B α_C_ helices are not well defined (Fig. 4c,d). The H/D exchange experiments showed that the amide protons in both helices were exchanged relatively rapid as compared with the amide protons in the α_2_, α_3_ and α_C_ helices of H2A and the α_1_, α_2_ and α_3_ helices of H2B. In the nucleosome, the H2A α_1_ helix region interacts with DNA and another H2A molecule, as shown in Supplementary Fig. S10, which stabilizes the location of the helix. Without either DNA or another H2A, the location of the H2A α_1_ helix may fluctuate. In addition, the H2B α_C_ helix interacts with the H2A α_N_ helix in the nucleosome, as shown in Supplementary Fig. S10; however, the H2A α_N_ helix becomes disordered in the isolated H2A-H2B heterodimer, and thus the location of the H2B α_C_ helix fluctuates.

In addition, H/D exchange experiments showed some elements of structure in the disordered C-terminal H2A tail around amino acids Val114 and the disordered N-terminal H2B tail around amino acid Lys11. In the nucleosome, amino acids Ala113, Val114 and Leu115 of H2A form a 3_10_ helix that interacts with the α_2_ helix of H3 and the L1 loop of H4. Although the model structure showed that the amino acids Ala113, Val114 and Leu115 of H2A did not form a 3_10_ helix in the isolated H2A-H2B, H/D exchange experiments suggested that this region has some propensity toward a helical structure.

Regarding the amide proton of Lys11 of H2B, in two of the 10 calculated structures, the amide proton interacts with Ser14 and Lys12. In addition, in the experiments of rapid exchange with water, Arg11, Ala12, Lys13 and Ala14 in the N-terminal disordered H2A tail showed slightly slower exchange as compared with other regions in the tail.

**Figure 8:**
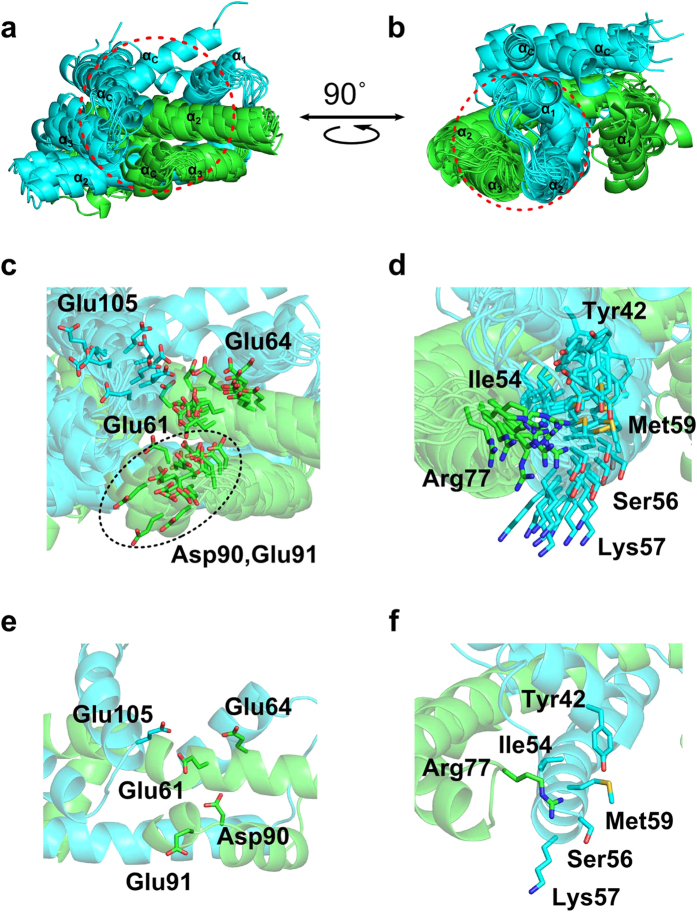
Interaction surfaces of the isolated H2A-H2B heterodimer. (**a**,**b**) Location of the two protein-binding regions of the H2A-H2B dimer: the acidic patch (**a**) and the histone chaperone binding region (**b**). Red dashed circles highlight the binding surfaces in the core region of the model structures of the isolated H2A-H2B heterodimer. (**c**,**d**) Close-up showing the major residues that interact with each partner in the binding regions shown in (**a**,**b**), respectively. (**e**,**f**) The two binding regions of the lowest core energy structures, corresponding to (**c**,**d**), respectively.

In the nucleosome, amino acids 27–34 of the H2B N-terminal tail—the so-called histone H2B repression domain (KKRKRSRK)—interacts with DNA^30^. This basic segment seems to adopt an extended string-like structure in the isolated H2A-H2B on the basis of our hetero-nuclear NOE experiments and CS-Rosetta calculation. This extended-like character seems to be important for the interaction with DNA gyres. Recently, the histone chaperone FACT, comprising Spt16 and Pob3, was found to bind to H2A-H2B primarily via the C-terminal acidic domains of Spt16 and Pob3^31^, suggesting that there is a common binding motif for H2A-H2B in three histone chaperones; namely, FACT (Spt16 and Pob3), ANP32E^13^ and Swr1^12^. We also identified two regions containing the binding motif for H2A-H2B in the C-terminal acidic domain (CTAD) of human nucleosome assembly protein 1 (hNAP1)^32^. The region of H2A-H2B that interacts with this binding motif is well defined in our isolated H2A-H2B structure, as shown in Fig. 8. However, the two regions in the hNAP1 CTAD bind to a single H2A-H2B heterodimer; thus, they seem to bind to two different regions of the same H2A-H2B heterodimer^32^. For some histone-interacting proteins, H2A-H2B provides an acidic patch^33^. This region is also well defined in our calculated structure, as shown in Fig. 8. However, these interactions remain to be investigated in further studies based on our present structure.

In summary, we have presented the first solution structure of the isolated human H2A-H2B heterodimer, including its flexible tail regions, resolved by NMR coupled with CS-Rosetta. This structure will provide insight into the dynamic functions and interactions of histone H2A-H2B in and out of the nucleosome.

## Methods

### Purification of human H2A and H2B

Recombinant human H2A and H2B were prepared as previously described^34^. We modified an existing pET-23b based vector, which encodes an N-terminal oct-histidine (His8) tag and Turbo3C protease cleavage site followed by Lumio^TM^ tag (Invitrogen). Proteins were expressed in *Escherichia coli* strain BL21 (DE3) star grown in LB medium. Each of the ^15^N-labeled or ^13^C/^15^N-labeled proteins was expressed in M9 minimal medium containing ^15^N-ammonium chloride with or without ^13^C-glucose. The ^2^H-labeled protein was expressed in 100% deuterated M9 minimal medium and the ^2^H/^13^C/^15^N-labeled protein was expressed in 100% deuterated M9 minimal medium containing ^15^N-ammonium chloride and ^13^C-glucose.

The harvested cells were re-suspended in Buffer A (50 mM Tris pH 8.0, 500 mM NaCl), lysed on ice by sonication, and centrifuged. The pellet was solubilized in Buffer B (50 mM Tris pH 8.0, 500 mM NaCl, 7 M guanidine hydrochloride). The protein solution was then applied to an immobilized-metal affinity chromatography (IMAC) column (BioRad) equilibrated with Buffer B, and His-tagged H2A or H2B was eluted by Buffer C (50 mM Tris–HCl pH 8.0, 500 mM NaCl, 3 M guanidine hydrochloride and 300 mM imidazole). The eluted His-tagged H2A or H2B was dialyzed against Buffer D (20 mM Tris pH 8.0 and 5 mM mercaptoethanol) and digested with Turbo3C protease (Accelagen) at 4 °C overnight. The protein solution was again loaded onto the IMAC column. Fractions passing through the column were concentrated and dialyzed against pure water. Lastly, the purified H2A or H2B was lyophilized.

### Preparation of the H2A-H2B heterodimer

Lyophilized H2A and H2B were mixed at a molar ratio of 1:1 and the H2A-H2B dimer was refolded by dialysis against Buffer E (20 mM Tris pH 8.0, 1 mM ethylene diamine tetraacetic acid (EDTA) and 2 M NaCl) followed by Buffer F (20 mM Tris pH 8.0, 1 mM EDTA and 1 M NaCl) at 4 °C. After dialysis, the sample solution was subjected to size exclusion chromatography (SEC) using a column of Superdex 200 pg (GE Healthcare) equilibrated with Buffer F at 4 °C; the eluted H2A-H2B dimer was stored at 4 °C.

### Chemical shifts of the H2A-H2B heterodimer in solutions

For NMR, a concentration of 0.1–0.3 mM H2A-H2B in 25 mM MES pH 6.0, 400 mM KCl dissolved in 90% H_2_O/10% D_2_O was used. The NMR experiments were performed at 20 °C on Bruker Avance 600-MHz and 800-MHz spectrometers, both with a 5-mm triple-resonance pulsed-field gradient cryoprobe. Chemical shifts were referenced to the chemical shift of 2,2-dimethyl-2-silapentane-5-sulfonate. The ^15^N and^13^C chemical shifts were referenced indirectly to 2,2-dimethyl-2-silapentane-5-sulfonate using the absolute frequency ratios.

Backbone and side chain resonances were assigned via the following experiments: TROSY-HN(CO)CACB, TROSY-HNCACB, TROSY-HN(CA)CO, TROSY-HNCO, HCCCONH, 2D TROSY-^1^H–^15^N HSQC and ^13^C HSQC. All NMR spectra were processed with the program NMRPipe^35^ and analyzed by the program Olivia (M. Yokochi, S. Sekiguchi & F. Inagaki, Hokkaido University, Sapporo, Japan).

### Structure calculation of H2A-H2B based on the chemical sifts

The solution structures of the isolated H2A-H2B heterodimer were modeled by the CS-Rosetta program^20^, which is a combination of the Rosetta program, the SPARTA program^21^ and MFR scripts^36^ and is able to generate model structures consistent with the observed chemical shifts. To obtain the overall structure of H2A-H2B, first the structure of the core regions of H2A (Val27-Leu96) and H2B (Ser38-Lys125) connected by a random coil (Gly)_16_ poly-glycine linker was modeled by the CS-Rosetta-AbinitioRelax protocol. The poly-glycine linker was then removed, the N-terminal and C-terminal H2A tails and N-terminal H2B tail were connected to the corresponding core structure, and the all-tail connected structure was modeled by CS-Rosetta using the FloppyTail protocol (CS-Rosetta-FloppyTail). All of the 1,221 chemical shift values observed from the HN, Hα, N, C’ Cα and Cβ signals were used in modeling.

The H2A-H2B core structure obtained from the CS-Rosetta-AbinitioRelax protocol was modeled as follows. (1) Based on the chemical shift values observed, 3-residue and 9-residue fragment libraries were generated from known protein structures. (2) In total, 10,000 models were generated by the Rosetta Monte Carlo fragment assembly method^22^ using the fragment libraries. (3) For each model generated, all-atom Rosetta energies^20^ were rescored to CS-Rosetta energy^20^ according to:


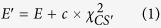


where *c* is a weighting factor set to 0.25 and the 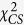 value indicates the reproducibility of the observed chemical shifts as follows:


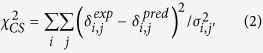


where 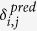 is the backbone chemical shift value of atom type *i* (HN, Hα, N, Cα, Cβ and C′) from the all-atom model for a given residue *j*, which is predicted by the SPARTA program^21^; 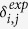 is the backbone chemical shift value observed from NMR experiments; and 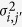 is the uncertainty of 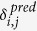. (4) Based on plots of CS-Rosetta energy versus root-mean-square-deviation (RMSD) values for Cα atoms in the lowest CS-Rosetta energy model, we selected the 10 models with the lowest Cα-RMSD from the 20 models with the lowest CS-Rosetta energy.

After the (Gly)_16_ poly-glycine linker was removed from each of the 10 selected structures of the H2A-H2B core, the flexible tails were generated by using the CS-Rosetta-FloppyTail protocol. The tails were modeled in the following order: the H2A N-terminal tail, Gly(-3)-Pro26, followed by the H2A C-terminal tail, Leu97-Lys129 and then the H2B N-terminal tail, Gly(-3)-Tyr37. Initial models of the flexible tails were attached to the core via the MODELLER program version 9.14^37^. Next, for each of the three flexible tails, FloppyTail with the multiple flexible linker mode was used to generate 10,000 models for each of the 10 selected core structures in conjunction with 3-residue and 9-residue fragment libraries for the tails generated from known protein structures by the CS-Rosetta program. We selected one model with the lowest 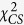 values calculated by the SPARTA program for each of the core structures to ultimately determine 10 structures of the isolated H2A-H2B heterodimer. In all of the above processes, serious structural defects were checked by using the WHAT_CHECK program^38^.

### Hydrogen-deuterium exchange experiments of H2A-H2B

The reference 2D TROSY-^1^H-^15^N HSQC spectrum of hydrogen-deuterium exchange was obtained from 0.4 mM H2A-H2B dissolved in 25 mM MES, 400 mM KCl pH 6.0 (90% H_2_O/10% D_2_O) at 293 K by using a Bruker Avance III HD 950-MHz spectrometer. The reference sample was recovered, lyophilized and reconstituted in the same volume of D_2_O as the previous volume of H_2_O. Immediately after reconstitution, 2D TROSY-^1^H–^15^N HSQC spectra of the sample were repeatedly recorded. All spectra were processed by NMRPipe and analyzed by the program Olivia (M. Yokochi, S. Sekiguchi & F. Inagaki, Hokkaido University, Sapporo, Japan).

### Fast hydrogen exchange experiments

HET^ex^ -BEST-TROSY^26^ experiments with relaxation times of 566, 878 and 1,659 ms were conducted by using a Bruker Avance III HD 950-MHz spectrometer with and without water saturation. The water signal strengths were measured by ^1^H-^15^N BEST-TROSY at each relaxation time using the small flip angle reading pulse. The *k*_*ex*_ value was obtained from equation (3,4) by using the signal intensities without water saturation, 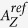 and those with water saturation, 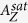, depending on the relaxation time, *d*_*relax*_, as follows:


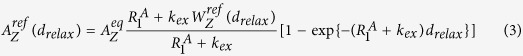



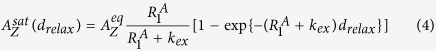


where 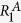 is a longitudinal relaxation rate of each amide proton, 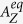 is the signal intensity at equilibrium, and 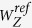 is the water intensity. The *k*_*ex*_ value was obtained by the least square fitting function of Gnuplot.

### Hetero-nuclear NOE experiments

{^1^H}-^15^N hetero-nuclear NOE^27^ experiments were performed on ^15^N-labeled H2A-H2B by using a Bruker Avance III HD 700-MHz spectrometer and TROSY type pulse sequence. Before NOE, ^1^H signals were saturated by the successive irradiation of 120-degrees pulses with 5-ms intervals for 5 seconds, and the intensities of the irradiated signals were compared with those of the un-irradiated signals.

## Additional Information

**Accession codes:** The structure and assigned chemical shifts for H2A-H2B have been deposited in the Protein Data Bank under accession code 2RVQ and Biological Magnetic Resonance Data Bank under accession code 11609.

**How to cite this article**: Moriwaki, Y. *et al*. Solution structure of the isolated histone H2A-H2B heterodimer. *Sci. Rep.* **6**, 24999; doi: 10.1038/srep24999 (2016).

## Supplementary Material

Supplementary Information

## References

[b1] Luger K. Structure and dynamic behavior of nucleosomes. Curr. Opin. Genet. Dev. 13, 127–135 (2003). 12672489 10.1016/s0959-437x(03)00026-1

[b2] Akey C. W. & Luger K. Histone chaperones and nucleosome assembly. Curr. Opin. Struct. Biol. 13, 6–14 (2003). 12581654 10.1016/s0959-440x(03)00002-2

[b3] De Koning L., Corpet A., Haber J. E. & Almouzni G. Histone chaperones: an escort network regulating histone traffic. Nat. Struct. Mol. Biol. 14, 997–1007 (2007). 17984962 10.1038/nsmb1318

[b4] Eitoku M., Sato L., Senda T. & Horikoshi M. Histone chaperones: 30 years from isolation to elucidation of the mechanisms of nucleosome assembly and disassembly. Cell. Mol. Life Sci. 65, 414–444 (2008). 17955179 10.1007/s00018-007-7305-6 PMC11131869

[b5] Luger K., Mäder A. W., Richmond R. K., Sargent D. F. & Richmond T. J. Crystal structure of the nucleosome core particle at 2.8 Å resolution. Nature 389, 251–260 (1997). 9305837 10.1038/38444

[b6] White C. L., Suto R. K. & Luger K. Structure of the yeast nucleosome core particle reveals fundamental changes in internucleosome interactions. EMBO. J. 20, 5207–5218 (2001). 11566884 10.1093/emboj/20.18.5207 PMC125637

[b7] Davey C. A., Sargent D. F., Luger K., Maeder A. W. & Richmond T. J. Solvent mediated interactions in the structure of the nucleosome core particle at 1.9 Å resolution. J. Mol. Biol. 319, 1097–1113 (2002). 12079350 10.1016/S0022-2836(02)00386-8

[b8] Harp J. M., Hanson B. L., Timm D. E. & Bunick G. J. Asymmetries in the nucleosome core particle at 2.5 Å resolution. Acta Crystallogr. D Biol. Crystallogr. 56, 1513–1534 (2000). 11092917 10.1107/s0907444900011847

[b9] Tsunaka Y., Kajimura N., Tate S. & Morikawa K. Alteration of the nucleosomal DNA path in the crystal structure of a human nucleosome core particle. Nucleic Acids Res. 33, 3424–3434 (2005). 15951514 10.1093/nar/gki663 PMC1150222

[b10] Hondele M. . Structural basis of histone H2A-H2B recognition by the essential chaperone FACT. Nature 499, 111–114 (2013). 23698368 10.1038/nature12242

[b11] Zhou Z. . NMR structure of chaperone Chz1 complexed with histones H2A.Z-H2B. Nat. Struct. Mol. Biol. 15, 868–869 (2008). 18641662 10.1038/nsmb.1465 PMC2574748

[b12] Hong J. . The catalytic subunit of the SWR1 remodeler is a histone chaperone for the H2A.Z-H2B dimer. Mol. Cell 53, 498–505 (2014). 24507717 10.1016/j.molcel.2014.01.010 PMC3940207

[b13] Obri A. . ANP32E is a histone chaperone that removes H2A.Z from chromatin. Nature 505, 648–653 (2014). 24463511 10.1038/nature12922

[b14] Berger S. L. The complex language of chromatin regulation during transcription. Nature 447, 407–412 (2007). 17522673 10.1038/nature05915

[b15] Downs J. A., Nussenzweig M. C. & Nussenzweig A. Chromatin dynamics and the preservation of genetic information. Nature 447, 951–958 (2007). 17581578 10.1038/nature05980

[b16] Felsenfeld G. & Groudine M. Controlling the double helix. Nature 421, 448–453 (2003). 12540921 10.1038/nature01411

[b17] Strahl B. D. & Allis C. D. The language of covalent histone modifications. Nature 403, 41–45 (2000). 10638745 10.1038/47412

[b18] Jenuwein T. & Allis C. D. Translating the histone code. Science 293, 1074–1080 (2001). 11498575 10.1126/science.1063127

[b19] Margueron R., Trojer P. & Reinberg D. The key to development: interpreting the histone code? Curr. Opin. Genet. Dev. 15, 163–176 (2005). 15797199 10.1016/j.gde.2005.01.005

[b20] Shen Y. . Consistent blind protein structure generation from NMR chemical shift data. Proc. Natl. Acad. Sci. USA 105, 4685–4690 (2008). 18326625 10.1073/pnas.0800256105 PMC2290745

[b21] Shen Y. & Bax A. Protein backbone chemical shifts predicted from searching a database for torsion angle and sequence homology. J. Biomol. NMR 38, 289–302 (2007). 17610132 10.1007/s10858-007-9166-6

[b22] Lange O. F. & Baker D. Resolution-adapted recombination of structural features significantly improves sampling in restraint-guided structure calculation. Proteins 80, 884–895 (2012). 22423358 10.1002/prot.23245 PMC3310173

[b23] Simons K. T., Kooperberg C., Huang E. & Baker D. Assembly of protein tertiary structures from fragments with similar local sequences using simulated annealing and Bayesian scoring functions. J. Mol. Biol. 268, 209–225 (1997). 9149153 10.1006/jmbi.1997.0959

[b24] Kleiger G., Saha A., Lewis S., Kuhlman B. & Deshaies R. J. Rapid E2-E3 assembly and disassembly enable processive ubiquitylation of cullin-RING ubiquitin ligase substrates. Cell 139, 957–968 (2009). 19945379 10.1016/j.cell.2009.10.030 PMC2804849

[b25] Dempsey C. E. Hydrogen exchange in peptides and proteins using NMR spectroscopy. Prog. Nucl. Magn. Reson. Spectrosc. 39, 135–170 (2001).

[b26] Rennella E., Solyom Z. & Brutscher B. Measuring hydrogen exchange in proteins by selective water saturation in ^1^H-^15^N SOFAST/BEST-type experiments: advantages and limitations. J. Biomol. NMR 60, 99–107 (2014). 25173410 10.1007/s10858-014-9857-8

[b27] Clore G. M., Driscoll P. C., Wingfield P. T. & Gronenborn A. M. Analysis of the backbone dynamics of interleukin-1β using two-dimensional inverse detected heteronuclear ^15^N-^1^H NMR spectroscopy. Biochemistry 29, 7387–7401 (1990). 2223770 10.1021/bi00484a006

[b28] Shaytan A. K., Landsman D. & Panchenko A. R. Nucleosome adaptability conferred by sequence and structural variations in histone H2A-H2B dimers. Curr. Opin. Struct. Biol. 32, 48–57 (2015). 25731851 10.1016/j.sbi.2015.02.004 PMC4512853

[b29] Kabsch W. & Sander C. Dictionary of protein secondary structure: pattern recognition of hydrogen-bonded and geometrical features. Biopolymers 22, 2577–2637 (1983). 6667333 10.1002/bip.360221211

[b30] Parra M. A., Kerr D., Fahy D., Pouchnik D. J. & Wyrick J. J. Deciphering the roles of the histone H2B N-terminal domain in genome-wide transcription. Mol. Cell. Biol. 26, 3842–3852 (2006). 16648479 10.1128/MCB.26.10.3842-3852.2006 PMC1489011

[b31] Kemble D. J., McCullough L. L., Whitby F. G., Formosa T. & Hill C. P. FACT Disrupts Nucleosome Structure by Binding H2A-H2B with Conserved Peptide Motifs. Mol. Cell 60, 294–306 (2015). 26455391 10.1016/j.molcel.2015.09.008 PMC4620744

[b32] Ohtomo H. . C-terminal acidic domain of histone chaperone human NAP1 is an efficient binding assistant for histone H2A-H2B, but not H3-H4. Genes Cells 21, 252-263 (2016). 26841755 10.1111/gtc.12339

[b33] Kato H. . Architecture of the high mobility group nucleosomal protein 2-nucleosome complex as revealed by methyl-based NMR. Proc. Natl. Acad. Sci. USA 108, 12283–12288 (2011). 21730181 10.1073/pnas.1105848108 PMC3145696

[b34] Tanaka Y. . Expression and purification of recombinant human histones. Methods 33, 3–11 (2004). 15039081 10.1016/j.ymeth.2003.10.024

[b35] Delaglio F. . NMRPipe: a multidimensional spectral processing system based on UNIX pipes. J. Biomol. NMR 6, 277–293 (1995). 8520220 10.1007/BF00197809

[b36] Kontaxis G., Delaglio F. & Bax A. Molecular fragment replacement approach to protein structure determination by chemical shift and dipolar homology database mining. Methods Enzymol 394, 42–78 (2005). 15808217 10.1016/S0076-6879(05)94003-2

[b37] Sali A. & Blundell T. L. Comparative protein modelling by satisfaction of spatial restraints. J. Mol. Biol. 234, 779–815 (1993). 8254673 10.1006/jmbi.1993.1626

[b38] Hooft R. W., Vriend G., Sander C. & Abola E. E. Errors in protein structures. Nature 381, 272 (1996). 8692262 10.1038/381272a0

[b39] Schrödinger LLC PyMOL: The PyMOL Molecular Graphics System, Version 1.7.6.6. Schrödinger LLC, NY, USA. URL https://www.pymol.org/ (2015).

