# Supplementary material for: Solution structure of the isolated histone H2A-H2B heterodimer
Source: Sci Rep. 2016 May 16;6:24999. doi: 10.1038/srep24999 (PMC4867618; doi:10.1038/srep24999)
Supplement: Supplementary Information [file srep24999-s1.pdf]

Supplementary information

Solution structure of the isolated histone H2A-H2B heterodimer

Yoshihito Moriwaki, Tsutomu Yamane, Hideaki Ohtomo, Mitsunori Ikeguchi, Jun-ichi Kurita, Masahiko Sato, Aritaka Nagadoi, Hideaki Shimojo, and Yoshifumi Nishimura*

Graduate School of Medical Life Science, Yokohama City University, 1-7-29 Suehiro-cho, Tsurumi-ku, Yokohama 230-0045, JAPAN

* Correspondence to: Yoshifumi Nishimura

nisimura@tsurumi.yokohama-cu.ac.jp

Tel: +81-45-508-7211, FAX: +81-45-508-7360

Convergence of solution structures of the isolated H2A-H2B heterodimer modelled by CS-Rosetta protocols.

First, we examined the convergence of the core and the tail region of the solution structures of the isolated H2A-H2B heterodimer. For the core region, a total of 10,000 models were generated by the CS-Rosetta program[1] using the AbinitioRelax application[2] in Rosetta 3.5 (CS-Rosetta-AbinitioRelax protocol). In the CS-Rosetta method, the χ^2_{CS} value defined by eq. 2 in the Methods was used to measure the reproducibility of the observed chemical shift for a model. The distribution of the χ^2_{CS} values of all of the generated core region structures were shown in Figure S2a. In calculating the core region, 821 experimental chemical shift values from HN, Hα, N, C', Cα and Cβ signals were used, and the lowest 10 % of χ^2_{CS} values were less than 940. For these models, therefore, the average difference between the calculated and observed chemical shift values was about 1.07σ $\left(= \sqrt{940/821}\,\sigma\right)$, where σ is the uncertainty of the chemical shift prediction of the SPARTA program[3] (Figure S2a). Thus, these models were in good agreement with the observed chemical shift values. However, the models with low χ^2_{CS} were not always the models with low Rosetta energy (Figure S2b), because the Rosetta energy does not include the experimental chemical shift values. As a result, the Rosetta energies were rescored as CS-Rosetta energy by incorporating the χ^2_{CS} value, as defined by eq. 1 in the Methods. In Figure S2c, the 10% of models with lowest χ^2_{CS} showed low CS-Rosetta energies, as mentioned in the original CS-Rosetta study[1]. In addition, the convergence of the core-region models was confirmed in the plot of the CS-Rosetta energy versus Cα-RMSD from the lowest CS-Rosetta energy model (Figure S2d). We selected the 10 lowest Cα-RMSD models from the 20 lowest CS-Rosetta energy models.

For convergence of the flexible tails, 96 (H2A-N terminus), 157 (H2A-C terminus) and 182 (H2B-N terminus) chemical shift values were used, and the lowest 10 % of χ^2_{CS} values for the H2A N- and C-, and H2B N-termini

were less than 160, 300 and 340, respectively (Figure S3, S5 and S7). For these models, therefore, the average difference between the calculated and observed chemical shift values was about 1.29σ $\left(= \sqrt{160/96}\,\sigma\right)$, 1.38σ $\left(= \sqrt{300/157}\,\sigma\right)$ and 1.37σ $\left(= \sqrt{340/182}\,\sigma\right)$ for the H2A N- and C-, and H2B N-termini, respectively. Thus, the flexible-tail models generated by the CS-Rosetta-FloppyTail protocol were also highly consistent with the observed chemical shift values. Because of their flexibility, however, these tail models did not converge into a single structure, and exhibited various interactions with other parts of the structure. This feature is represented in the wide range of the Rosetta energy values of these tail structures (Figure S4, S6 and S8). Thus, to model with the flexible tail, we selected the structures with the lowest χ^2_{CS} values at each modeling stage without using the Rosetta energies.

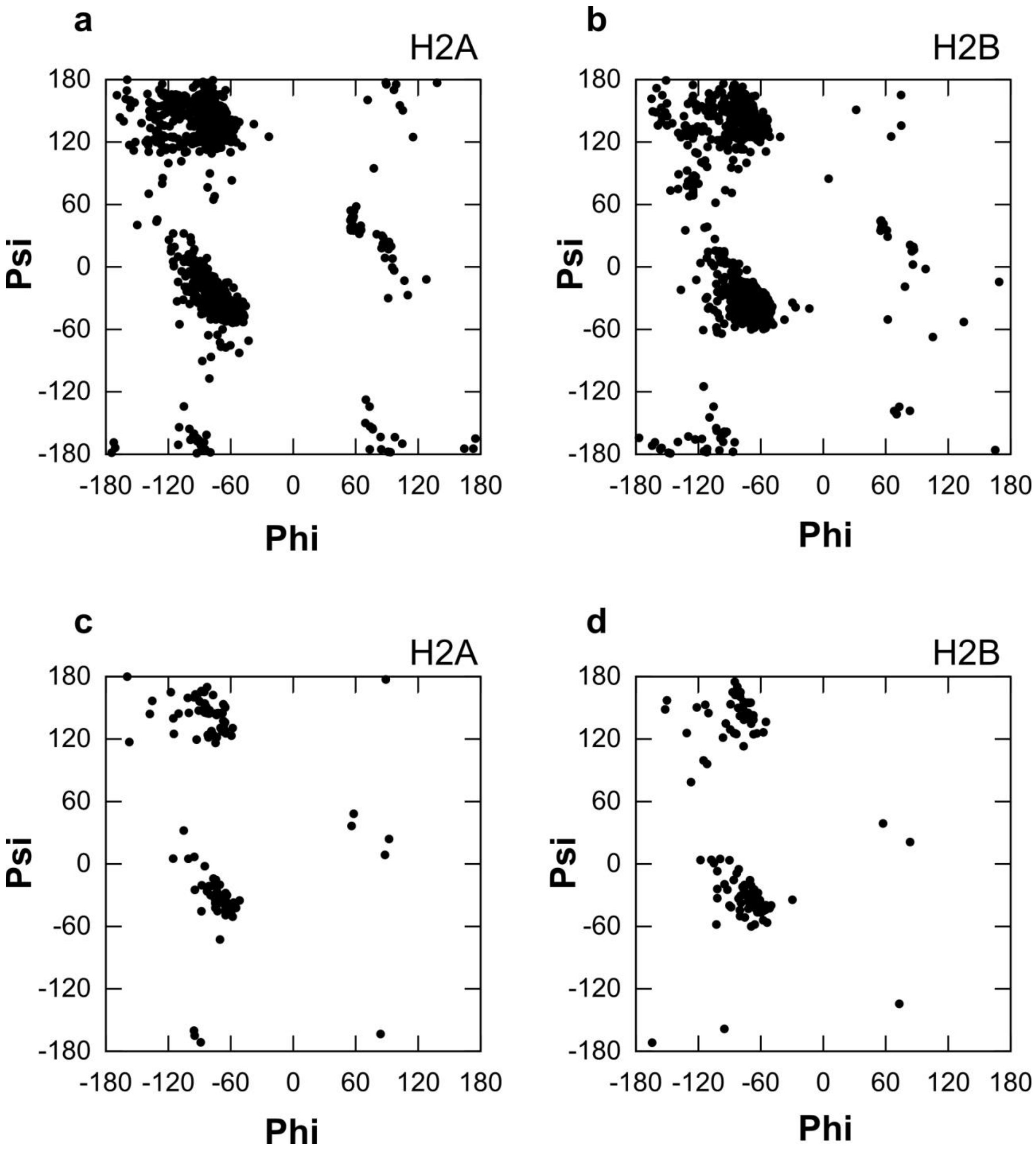

Supplementary Figure S1. Structural analysis of the H2A-H2B heterodimer. Ramachandran plots of H2A (**a**, **c**) and H2B (**b**, **d**) from the model structures of the isolated H2A-H2B heterodimer. The panel (**a**) and (**b**) are the plots of the ten model structures. The panel (**c**) and (**d**) are the plots of the structure with the lowest CS-Rosetta Energy score of the core region.

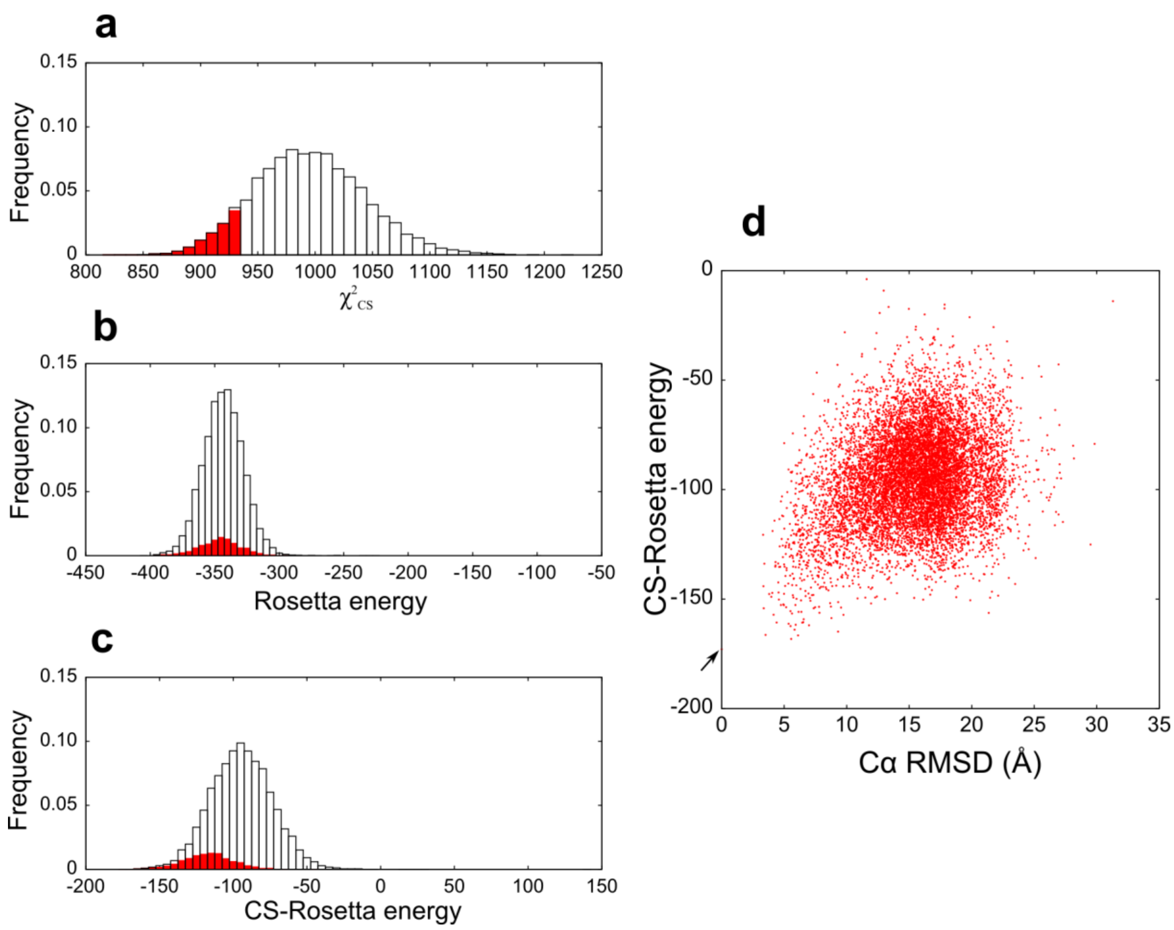

Supplementary Figure S2. Results of modeling of the core region of the isolated H2A-H2B heterodimer.

(**a-c**) Histograms of the χ^2_{CS} value (**a**), Rosetta energy (**b**) and CS-Rosetta energy (**c**) of the 10,000 structures generated in modeling of the core region of the H2A-H2B heterodimer. The red region indicates the lowest 10 % of χ^2_{CS} values. (**d**) Plots of CS-Rosetta energy versus Cα-RMSD relative to the lowest-energy model. The position of the lowest-energy model is shown by an arrow.

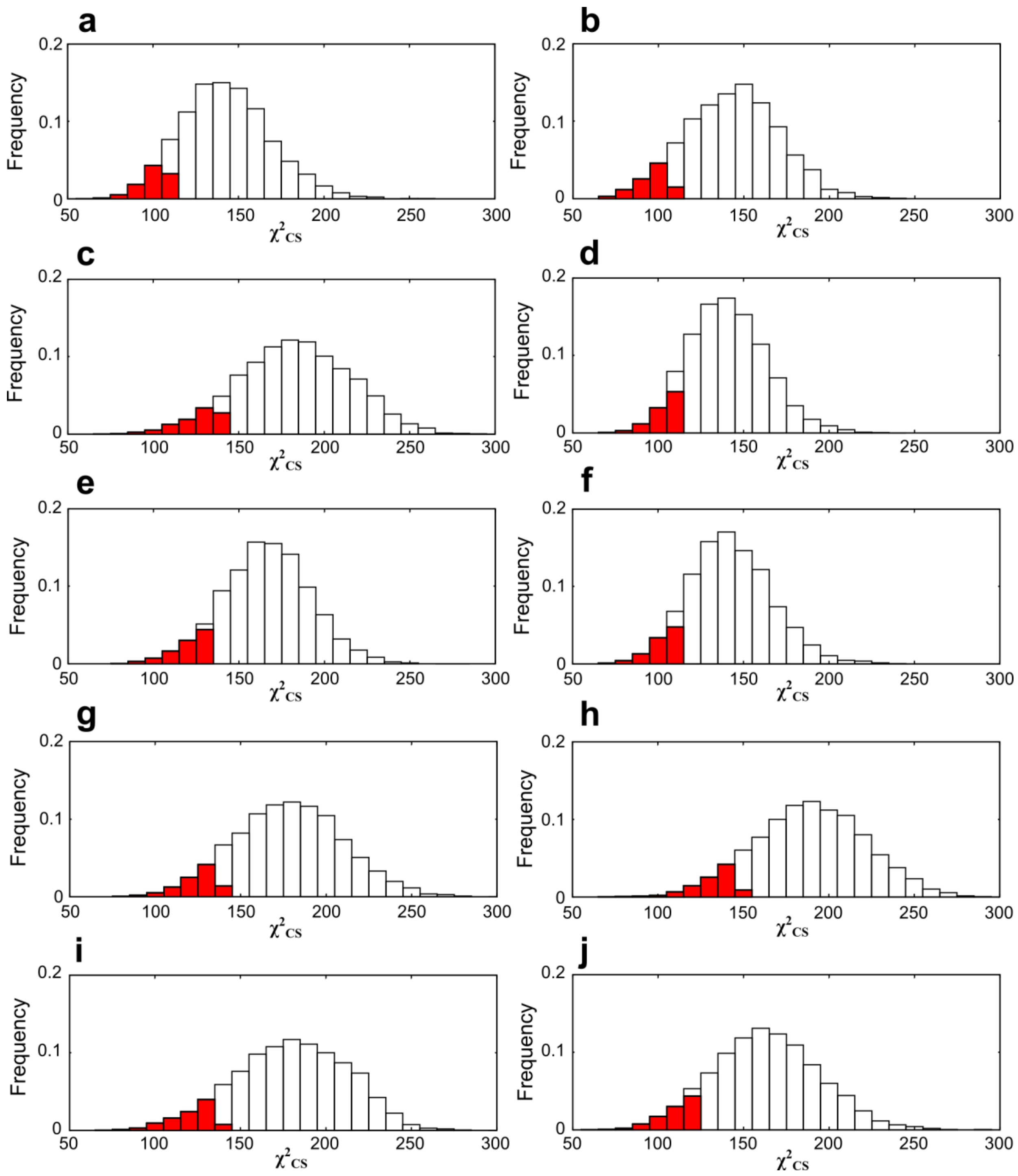

Supplementary Figure S3. Distribution of the χ^2_{CS} values in modeling of the H2A N-terminal tail region.

(**a-j**) Histograms of the χ^2_{CS} values of the 10,000 structures generated in modeling of the H2A N-terminal tail region to the 10 core structures of the H2A-H2B heterodimer. The red region indicates the lowest 10 % of χ^2_{CS} values.

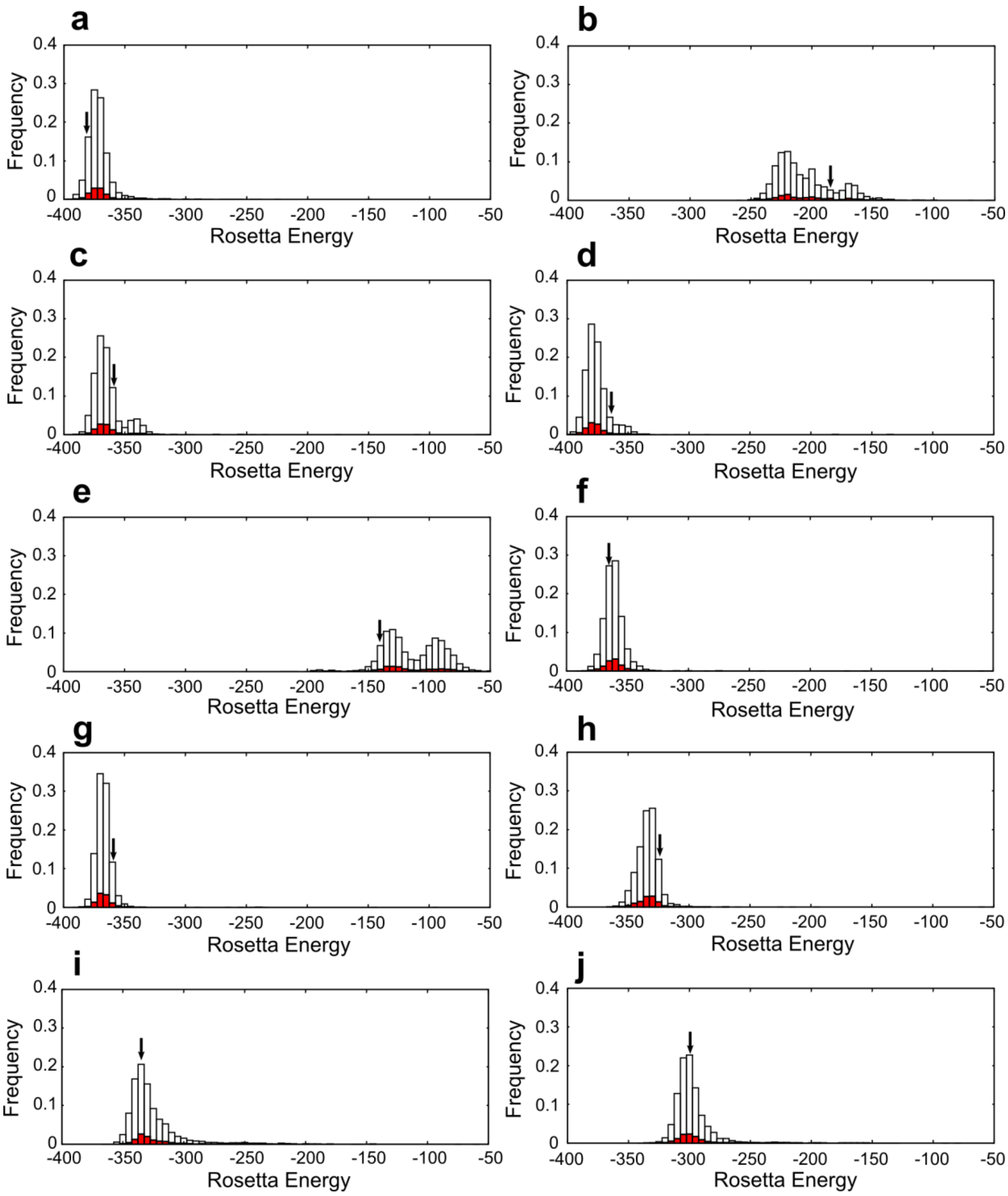

Supplementary Figure S4. Distribution of the Rosetta energy in modeling of the H2A N-terminal tail region.

(**a-j**) Histograms of the Rosetta energy of the 10,000 structures generated in modeling of the H2A N-terminal tail region to the 10 core structures of the H2A-H2B heterodimer. The red region indicates the lowest 10 % of χ^2_{CS} values; the Rosetta energy values of selected structures are shown by arrows.

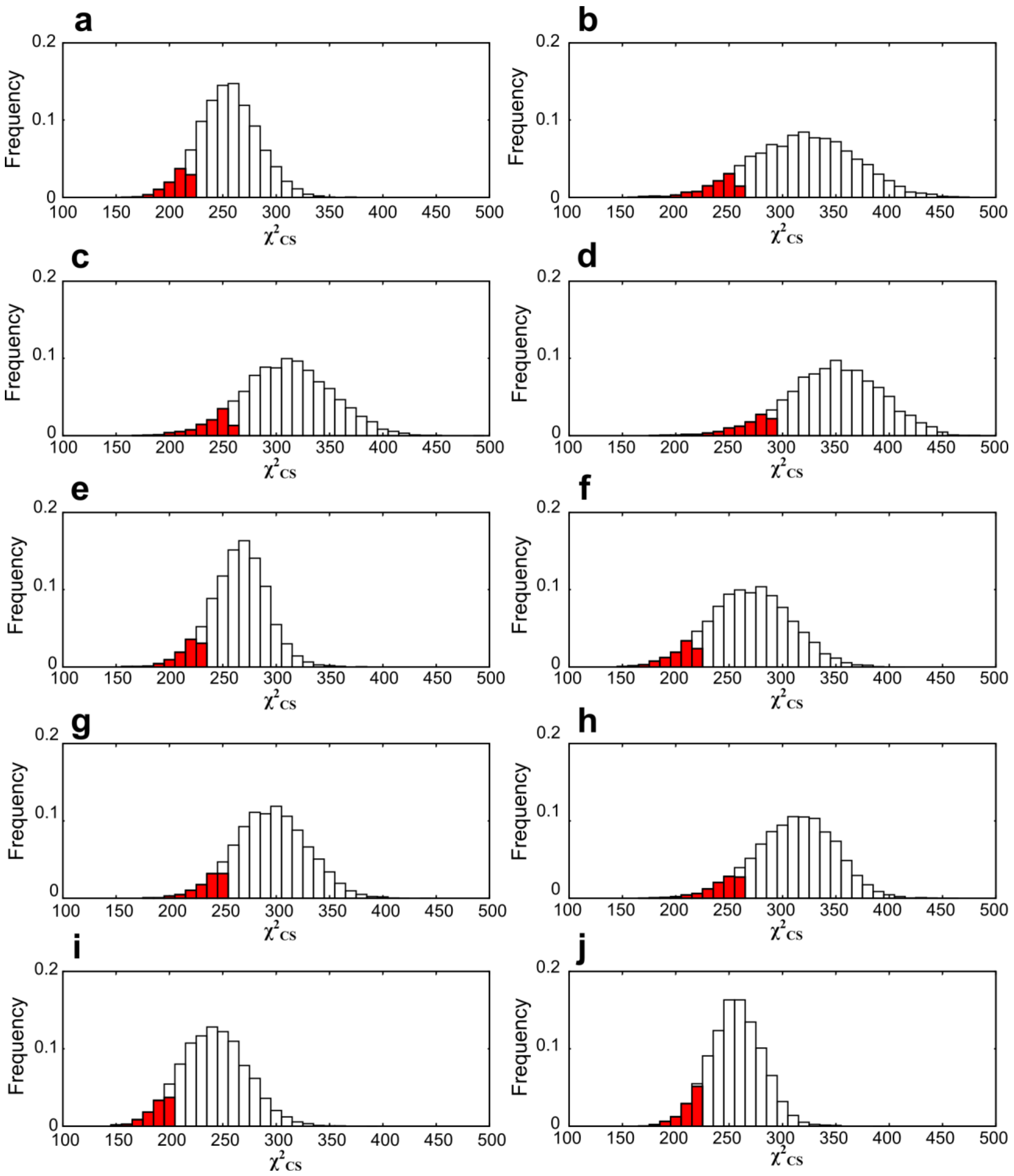

Supplementary Figure S5. Distribution of the χ^2_{CS} values in modeling of the H2A C-terminal tail region.

(**a-j**) Histograms of the χ^2_{CS} values of the 10,000 structures generated in modeling of the H2A C-terminal tail region to the 10 core structures of the H2A-H2B heterodimer. The red region indicates the lowest 10 % of χ^2_{CS} values.

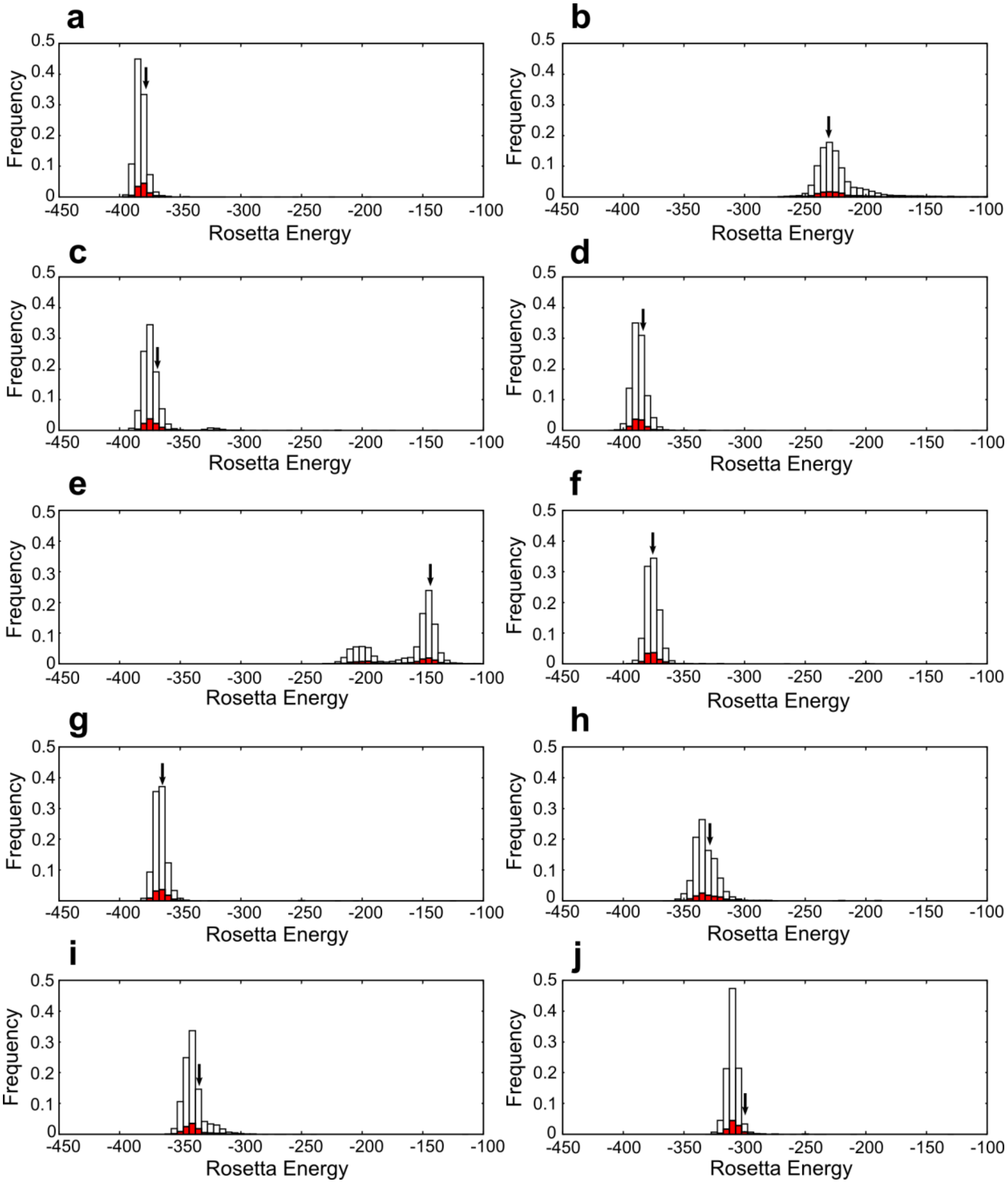

Supplementary Figure S6. Distribution of the Rosetta energy in modeling of the H2A C-terminal tail region.

(**a-j**) Histograms of the Rosetta energy of the 10,000 structures generated in modeling of the H2A C-terminal tail region to the 10 core structures of the H2A-H2B heterodimer. The red region indicates the lowest 10 % of χ^2_{CS} values; the Rosetta energy values of selected structures are shown by arrows.

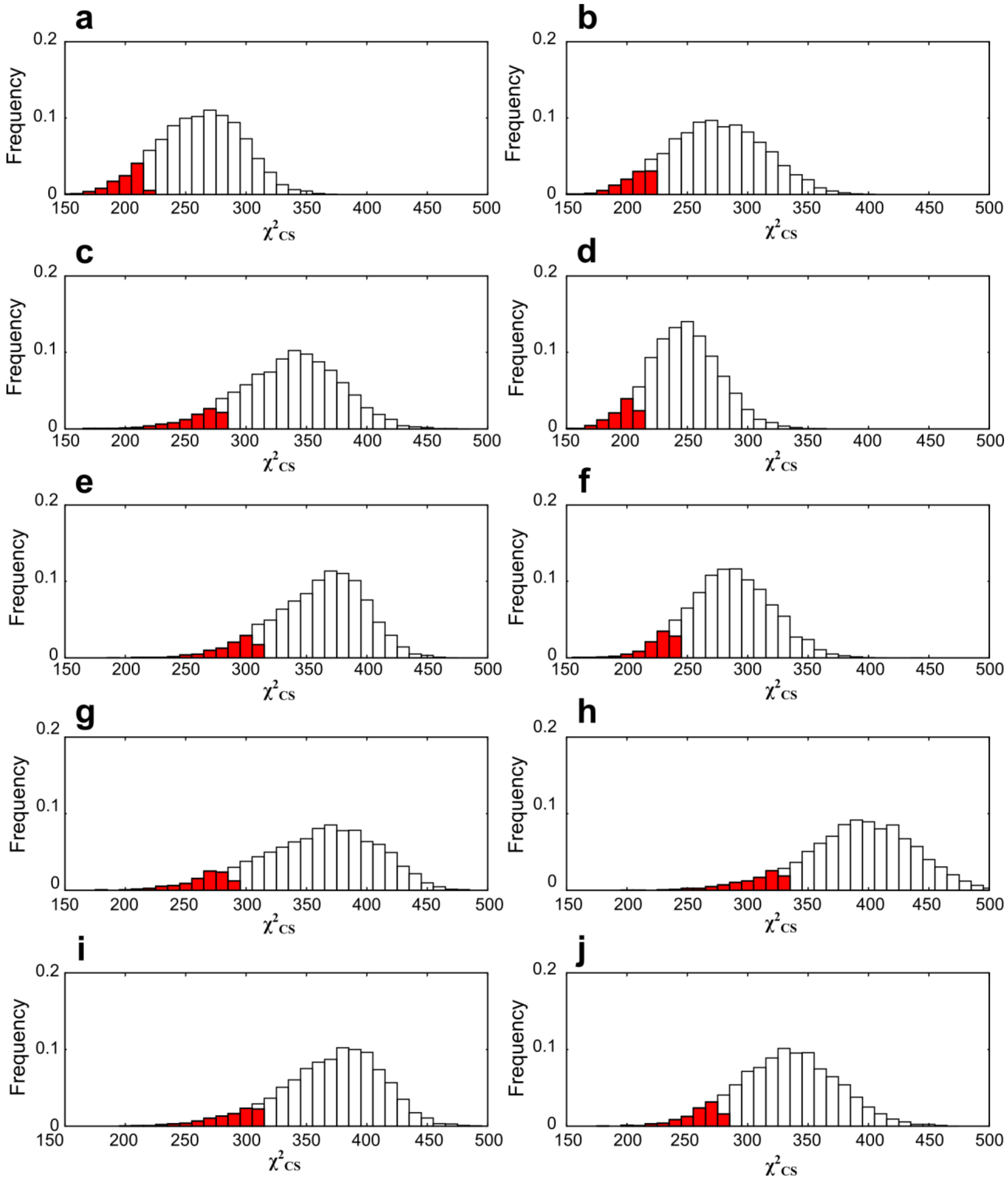

Supplementary Figure S7. Distribution of the χ^2_{CS} values in modeling of the H2B N-terminal tail region.

(**a-j**) Histograms of the χ^2_{CS} values of the 10,000 structures generated in modeling of the H2B N-terminal tail region to the 10 core structures of the H2A-H2B heterodimer. The red region indicates the lowest 10 % of χ^2_{CS} values.

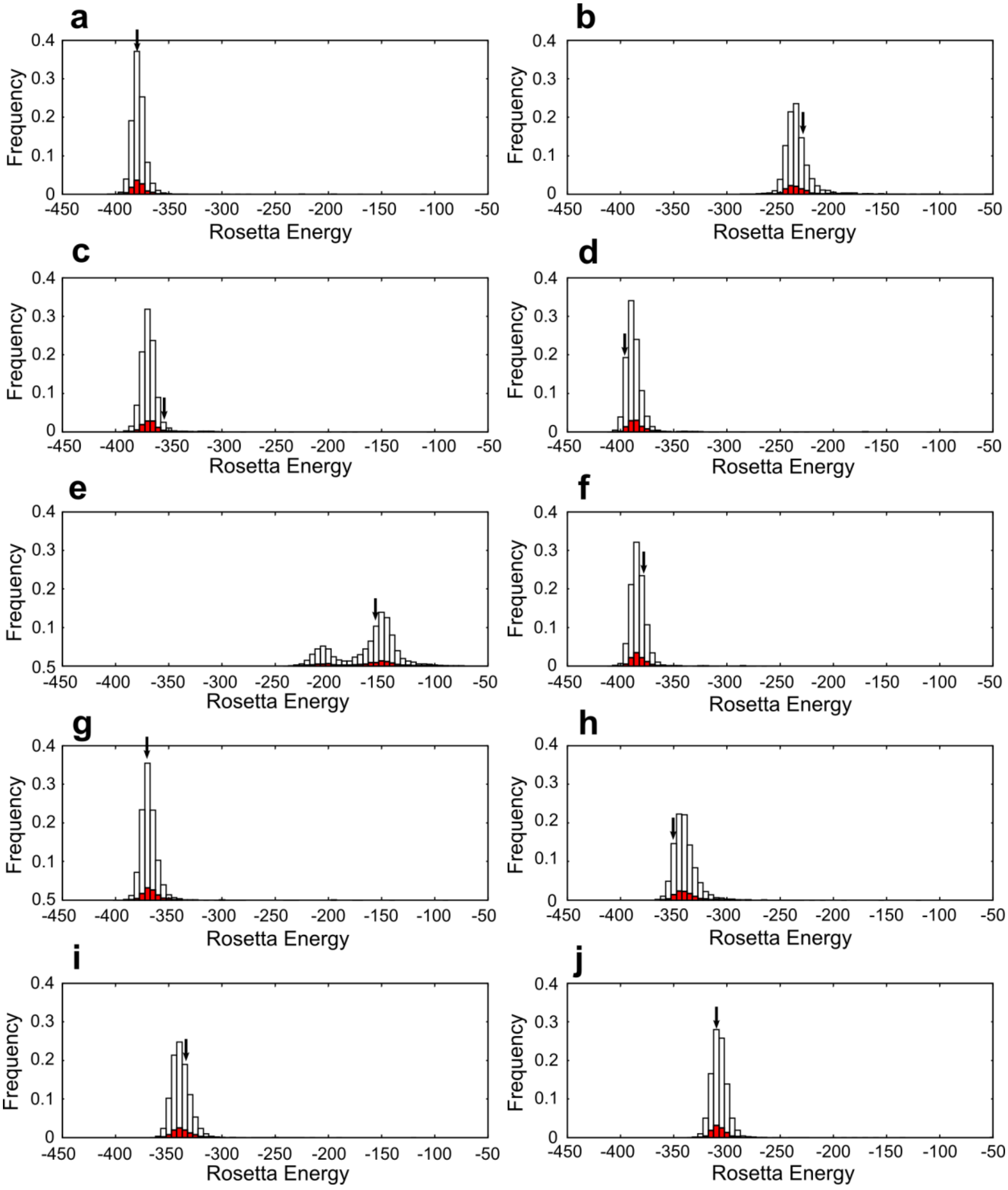

Supplementary Figure S8. Distribution of the Rosetta energy in modeling of the H2B N-terminal tail region.

(**a-j**) Histograms of the Rosetta energy of the 10,000 structures generated in modeling of the H2B N-terminal tail region to the 10 core structures of the H2A-H2B heterodimer. The red region indicates the lowest 10 % of χ^2_{CS} values; the Rosetta energy values of selected structures are shown by arrows.

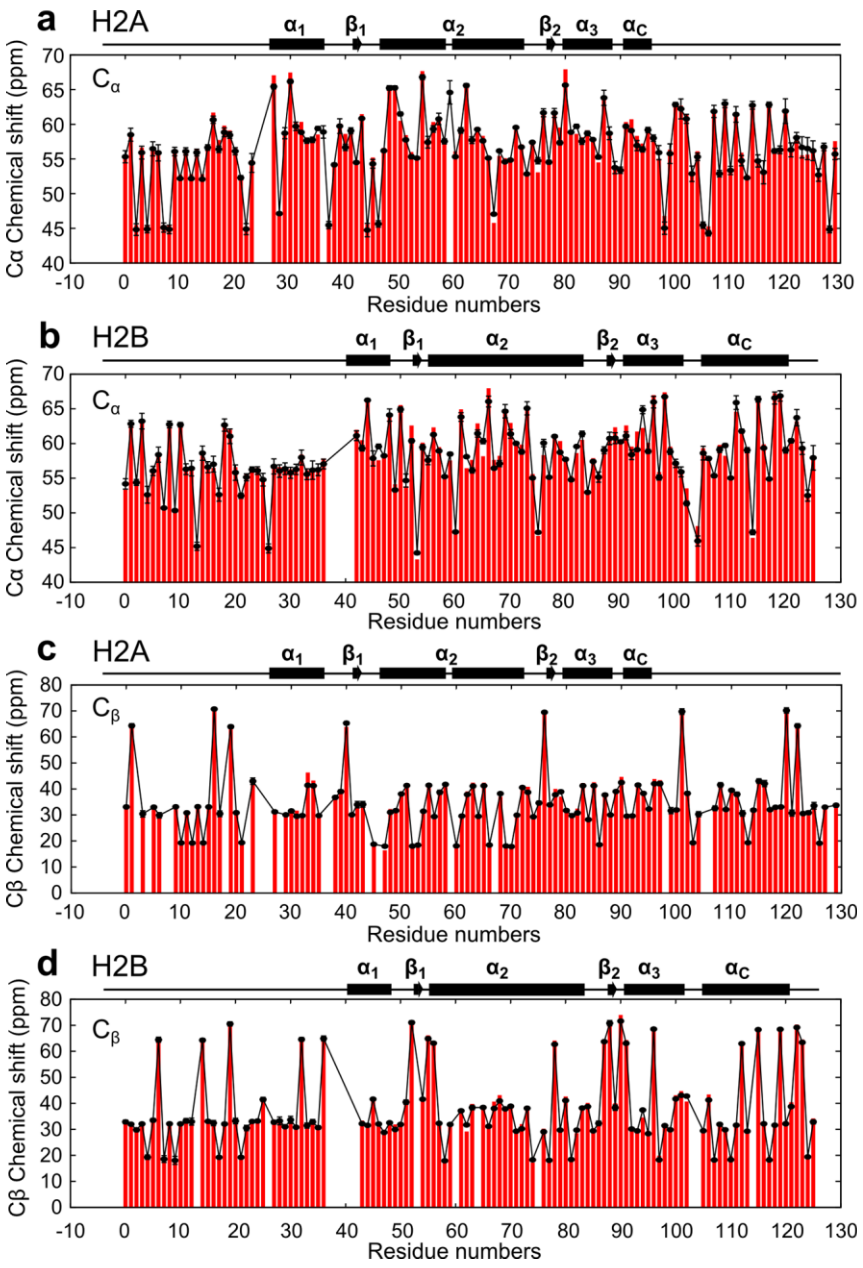

Supplementary Figure S9. Observed and calculated chemical shift values of the isolated H2A-H2B heterodimer. (**a**,**b**) Comparison of the Cα chemical shift values of H2A (**a**) and H2B (**b**) between the NMR measurement values (red bars) and the average of the calculated values of the modeled structures (black lines)**.** (**c**,**d**) Comparison of the Cβ chemical shift values of H2A (**c**) and H2B (**d**) between the NMR measurement values (red bars) and the average of the calculated values of the modeled structures (black lines). Residues that were missing in the NMR measurements are excluded from the panels.

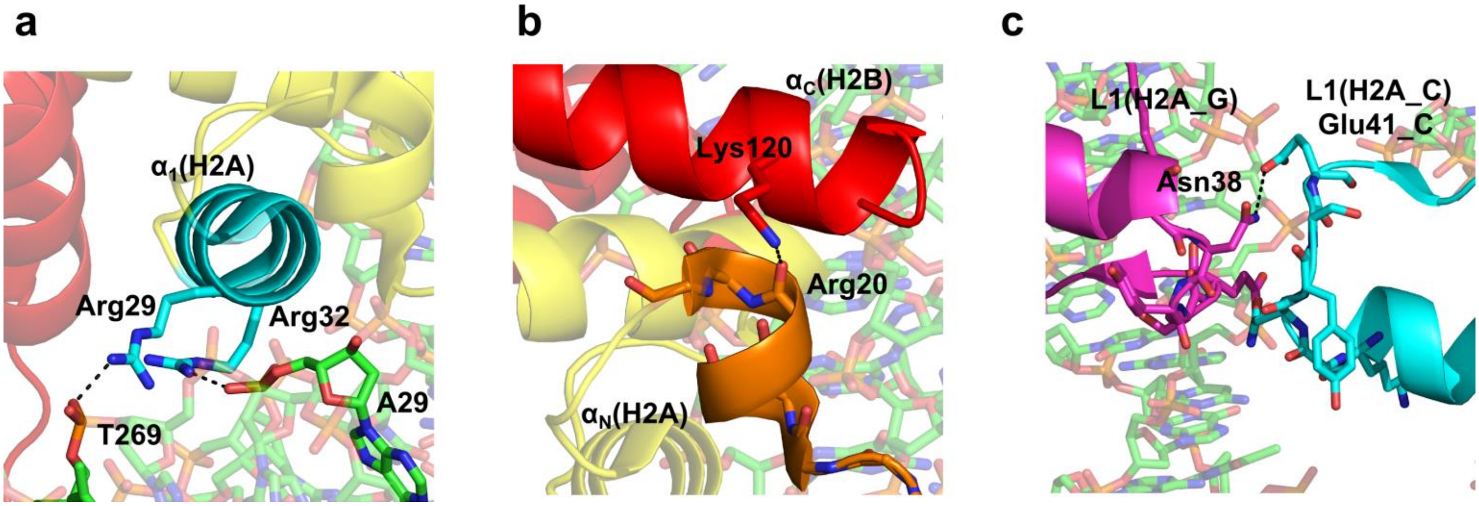

Supplementary Figure S10. Structural analysis of the H2A-H2B heterodimer in the nucleosome. (**a-c**) Interactions of H2A α_1 (**a**), H2B α_C (**b**) and the L1/L1 interface (**c**) in the crystal structure of the nucleosome (PDB ID: 3AFA). In (**a**), α_1 of H2A, and other region of H2A and H2B are shown in cyan, yellow and red, respectively. In (**b**), α_C of H2B and α_N of H2A are shown in red and orange, respectively. In (**c**), the L1 region, which is located between α_1 and β_1, is shown in stick notation, and the two H2A subunits are shown in magenta and cyan, respectively. Hydrogen bonds are shown as broken lines, and key residues are labeled. Images were drawn by PyMol[4].

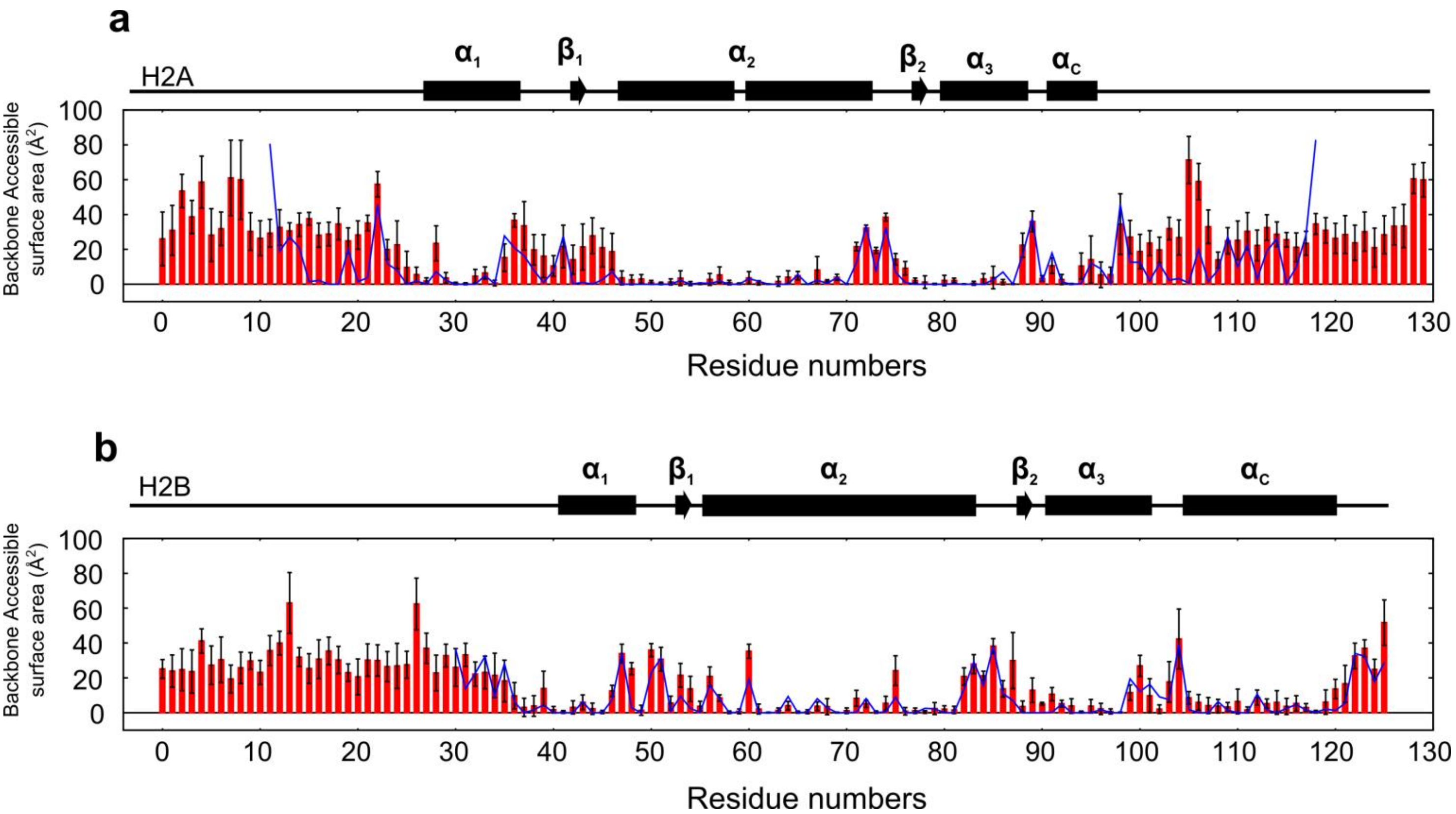

Supplementary Figure S11. Backbone accessible surface area of the isolated H2A-H2B heterodimer.

The accessible surface areas (ASAs) of each residue of the isolated H2A-H2B heterodimer are shown. (**a**) ASA of H2A; (**b**) ASA of H2B. The average values of the ASA of the model structures are shown as red bars with black error bars; and the ASAs of the H2A-H2B heterodimer in the nucleosome core are shown as blue lines. Secondary structural elements are indicated as boxes (α-helices) and arrows (β-strands). ASAs were calculated by the AREAIMOL program[5] in the CCP4 software suite[6].

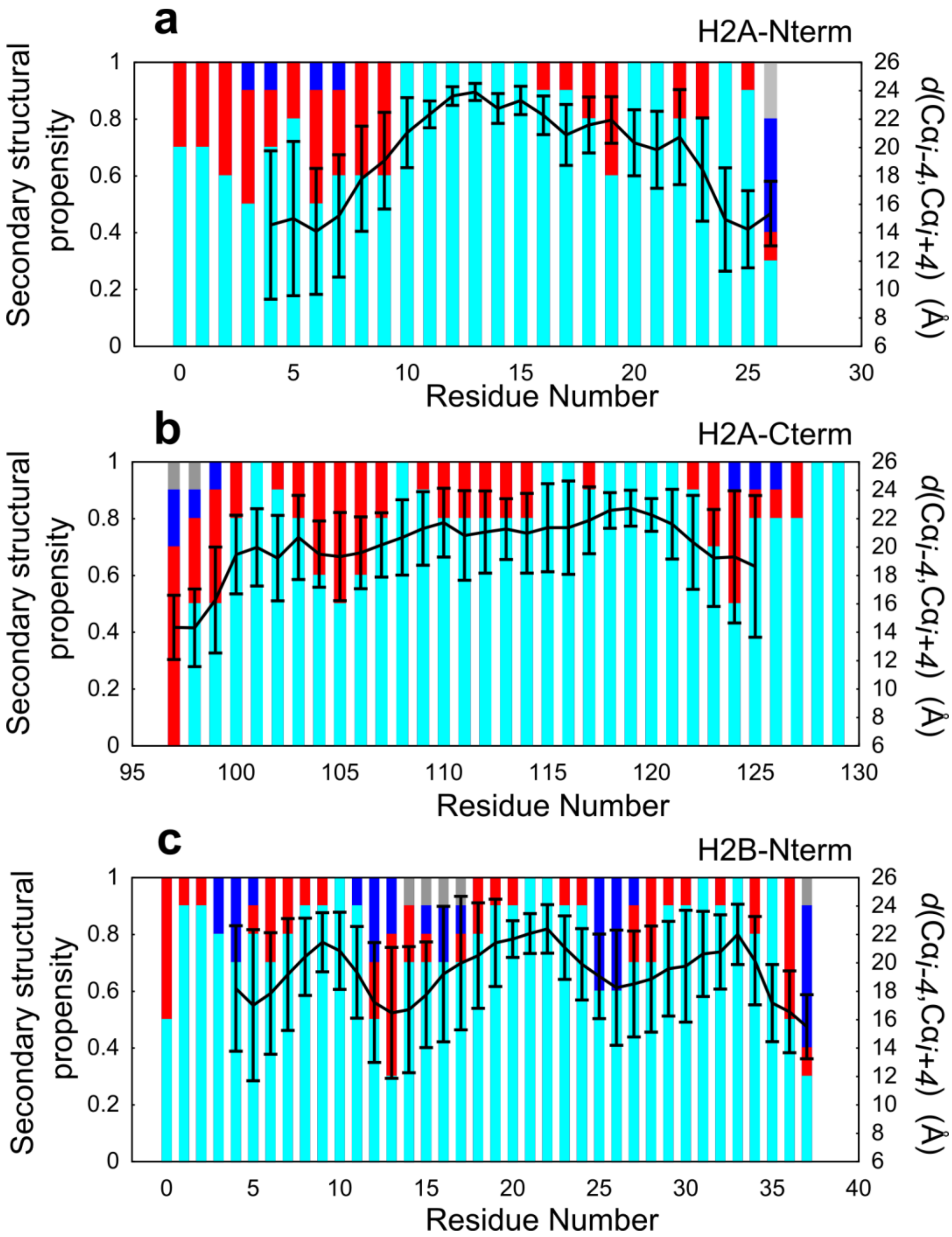

Supplementary Figure S12. Structural propensity of the long tail regions of the model structures of the isolated H2A-H2B heterodimer.

Secondary structural propensity and Cα distances of the long tail regions of the model structures of the isolated H2A-H2B heterodimer are shown. (**a**) H2A N-terminus; (**b**) H2A C-terminus; and (**c**) H2B N-terminus. Secondary structural propensity was calculated by the DSSP program[7], and turns ("T" in DSSP), bends ("S" in DSSP) , other

secondary structures ("H", "B", "E", "G", and "I" in DSSP) and non-assigned residues are shown in blue, red , gray and cyan, respectively. $d(C\alpha_{i\text{-}4}, C\alpha_{i+4})$, which is the distance between Cα atoms in the *i*-4 and *i*+4 residues, is shown by a black line with error bars.

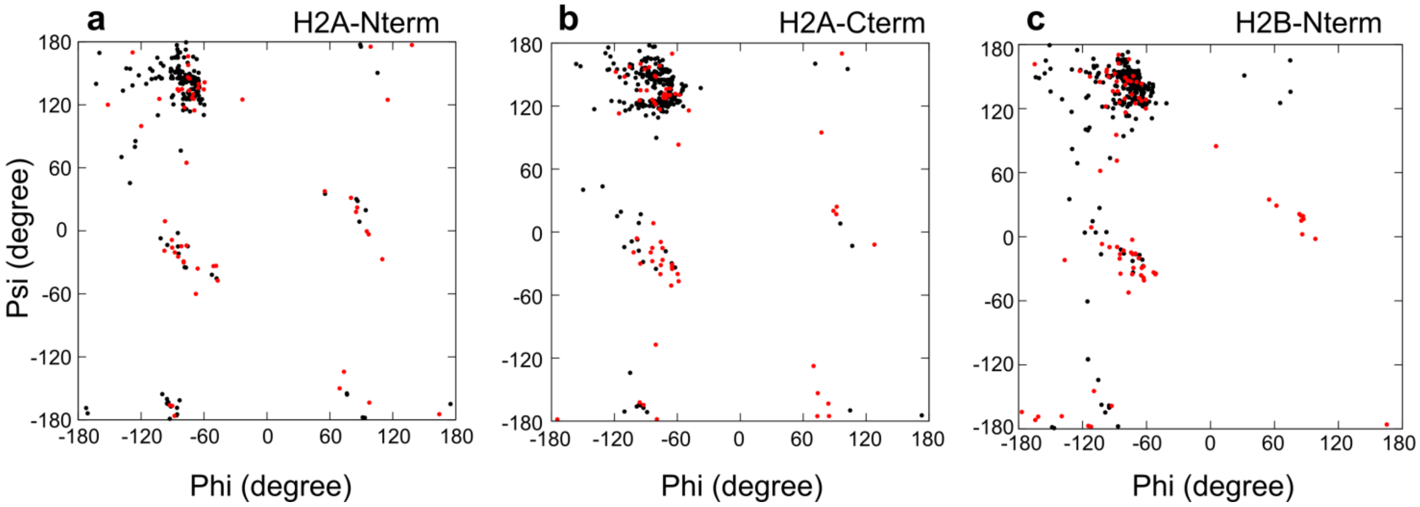

Supplementary Figure S13. Ramachandran plots of residues in the long tail regions of the model structures of the isolated H2A-H2B heterodimer.

Ramachandran plots of residues in the long tail regions are shown. (**a**) H2A N-terminus; (**b**) H2A C-terminus; and (**c**) H2B N-terminus. Turn ("T" in DSSP) and bends ("S" in DSSP) residues are plotted as red filled circles, and non-assigned residues are plotted as black filled circles.

References

1. Shen, Y. *et al.* Consistent blind protein structure generation from NMR chemical shift data. *Proc. Natl. Acad. Sci. U. S. A.* **105,** 4685-4690 (2008).
2. Simons, K. T., Kooperberg, C., Huang, E. & Baker, D. Assembly of protein tertiary structures from fragments with similar local sequences using simulated annealing and Bayesian scoring functions. *J. Mol. Biol.* **268,** 209-225 (1997).
3. Shen, Y. & Bax, A. Protein backbone chemical shifts predicted from searching a database for torsion angle and sequence homology. *J. Biomol. NMR* **38,** 289-302 (2007).
4. The PyMOL Molecular Graphics System, V. S., LLC.
5. Lee, B. & Richards, F. M. The interpretation of protein structures: estimation of static accessibility. *J. Mol. Biol.* **55,** 379-400 (1971).
6. Winn, M. D. *et al.* Overview of the CCP4 suite and current developments. *Acta Crystallogr. D Biol. Crystallogr.* **67,** 235-242 (2011).
7. Kabsch, W. & Sander, C. Dictionary of protein secondary structure: pattern recognition of hydrogen-bonded and geometrical features. *Biopolymers* **22,** 2577-2637 (1983).
